# Spatiotemporal parameters for energy efficient kilohertz-frequency nerve block with low onset response

**DOI:** 10.1186/s12984-023-01195-8

**Published:** 2023-06-05

**Authors:** Edgar Peña, Nicole A. Pelot, Warren M. Grill

**Affiliations:** 1 Department of Biomedical Engineering, Duke University, Room 1427, Fitzpatrick CIEMAS, 101 Science Drive Campus Box 90281, Durham, NC 27708 USA; 2 Department of Electrical and Computer Engineering, Duke University, Durham, NC USA; 3 Department of Neurobiology, Duke University School of Medicine, Durham, NC USA; 4 Department of Neurosurgery, Duke University School of Medicine, Durham, NC USA

**Keywords:** Kilohertz-frequency nerve block, Frequency effects, Waveform design, Onset response, Energy efficiency

## Abstract

**Background:**

Electrical nerve conduction block has great potential for treatment of disease through reversible and local inactivation of somatic and autonomic nerves. However, the relatively high energy requirements and the presence of undesired excitation at the onset of the kilohertz-frequency (KHF) signals used for block pose obstacles to effective translation. Frequency, electrode geometry, and waveform shape are known to influence block threshold and onset response, but available data provide a limited understanding of how to select these parameters to optimize nerve block.

**Methods:**

We evaluated KHF nerve block in rat tibial nerve across frequencies (5–60 kHz), electrode geometries (monopolar, bipolar, and tripolar), and waveform shapes. We present a novel Fourier-based method for constructing composite signals that systematically sample the KHF waveform design space.

**Results:**

The lowest frequencies capable of blocking (5–16 kHz) were not the most energy-efficient among the tested frequencies. Further, bipolar cuffs required the largest current and power to block, monopolar cuffs required the lowest current, and both tripolar and monopolar cuffs required the lowest power. Tripolar cuffs produced the smallest onset response across frequencies. Composite signals comprised of a first harmonic sinusoid at fundamental frequency (f_0_) superposed on a second harmonic sinusoid at 2f_0_ could block at lower threshold and lower onset response compared to the constituent sinusoids alone. This effect was strongly dependent on the phase of the second harmonic and on the relative amplitudes of the first and second harmonics. This effect was also dependent on electrode geometry: monopolar and tripolar cuffs showed clear composite signal effects in most experiments; bipolar cuffs showed no clear effects in most experiments.

**Conclusions:**

Our data provide novel information about block threshold and onset response at the boundary of frequencies that can block. Our results also show an interaction between spatial (cuff geometry) and temporal (frequency and waveform shape) parameters. Finally, while previous studies suggested that temporal parameters could reduce onset response only in exchange for increased block threshold (or vice versa), our results show that waveform shape influences KHF response in ways that can be exploited to reduce *both* energy and onset responses.

**Supplementary Information:**

The online version contains supplementary material available at 10.1186/s12984-023-01195-8.

## Background

Reversible block of nerve conduction using kilohertz-frequency (KHF) periodic signals has great potential as a therapy for a range of disorders [1]. Despite this clinical potential, translation is challenging because of the relatively high energy requirements compared to nerve stimulation and because of transient excitation that occurs at the initiation of KHF signals (i.e., onset response). High energy requirements limit the battery life of implantable devices and could exceed the output available from implantable pulse generators, while onset response can cause undesired effects when action potential volleys reach the nerve’s upstream or downstream targets (e.g., target muscles/organs, brainstem). Therefore, parameters that achieve KHF nerve block with minimal energy and onset response are of great translational interest.

Electrical block is typically achieved with periodic waveforms (sinusoids, square, rectangular) applied at kilohertz frequencies (> 5 kHz) to a target nerve through cuff electrodes containing one to three contacts (monopolar, bipolar, tripolar). Several studies measured the effects of these temporal and spatial parameters on the efficiency and onset response of KHF nerve block. Higher frequencies had lower onset response and higher block thresholds [2–5]. Waveform shape had no clear effect on onset response, although block thresholds were lowest for square waves and highest for triangular waves or low duty cycle rectangular waves [4–6]. Inter-contact spacing and contact circumferential lengths could influence block threshold and onset response, although only bipolar cuffs parameters were considered [7–9].

Despite the insights from previous studies, important questions remain regarding the spatial and temporal parameters to achieve KHF nerve block efficiently and with low onset response. For example, it is not apparent whether the most efficient blocking frequency is the lowest frequency that achieves block because characterizations of block threshold at the frequency boundaries of nerve block were not reported. Further, an infinite number of periodic waveforms are possible beyond the previously explored square, rectangular, and triangular waveforms; thus, the ability of alternative waveform shapes to modify KHF nerve block characteristics remains underexplored. Finally, although a number of different cuff geometries were used in prior literature (for overview tables, see [6, 7, 9]), those studies did not investigate the effects of the number of contacts nor, crucially, the interaction between temporal parameters (i.e., frequency, waveform shape) and electrode geometry.

We conducted in vivo experiments on rat tibial nerve to identify the frequency, waveform shape, and number of contacts that blocked with minimum amplitude, minimum power, and minimum onset response. Our goals were to evaluate the following metrics across multiple cuff geometries: (1a) nerve-specific *minimum blocking frequency*, (1b) block threshold current and power across frequencies, (1c) onset response across frequencies; (2a) block threshold rms across waveforms shapes; and (2b) onset response across waveform shapes. We developed a new method that leveraged Fourier analysis to tune systematically the waveform shape. We conducted experiments using monopolar, bipolar, and tripolar nerve cuffs to evaluate electrode geometry effects on efficiency and onset response, and to quantify interactions between the effects of waveform and cuff geometry.

## Methods

We used in vivo experimental measurements as in our previous KHF studies [3, 4] to quantify the effects of frequency, waveform shape, and cuff geometry on block of the rat tibial nerve. We aimed to understand the impact of these parameters on the efficiency (i.e., block threshold) and onset response. First, we measured block threshold and onset responses across frequencies to identify the minimum frequency that produced block. We then measured the block threshold and onset responses across a range of periodic waveforms synthesized by superposition of two sinusoids of differing frequency, amplitude, and phase. We conducted these measurements with monopolar, bipolar, and tripolar cuffs to evaluate the effect of number of contacts as well as the interactions of frequency and waveform shape with the number of contacts. We used MATLAB R2018a (Mathworks; Natick, MA) for all data analyses, plotting, and statistics.

### In vivo electrical block of the rat tibial nerve

**Fig. 1:**
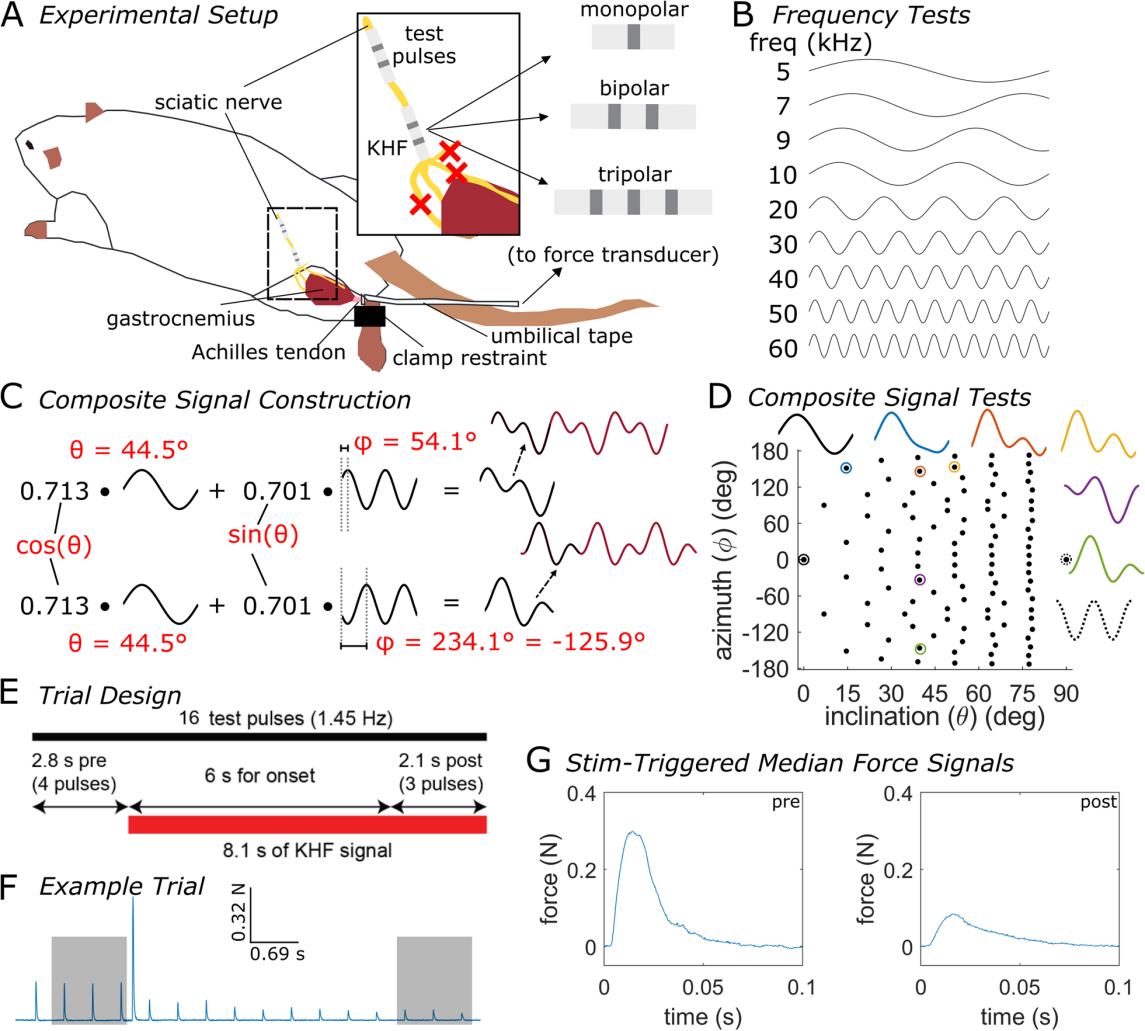
Overview of experimental methods to measure the characteristics of KHF block. **A** Experimental preparation of tibial nerve block in an anesthetized rat. Red crosses indicate transected nerve branches. The electrodes used for KHF signals were either monopolar, bipolar, or tripolar (one cuff type per rat). **B** Initial set of frequencies applied during frequency tests in each experiment. **C** Composite signal construction for composite signal tests. We constructed each composite signal by superposing a sinusoid of frequency f_0_ with a sinusoid of frequency 2f_0_, where f_0_ was a nerve-specific fundamental frequency. We scaled the f_0_ and 2f_0_ sinusoids by different relative amplitudes (amplitudes of sin(θ) and cos(θ), respectively, where θ is the inclination). We shifted the phase of the second harmonic (azimuth, φ) to produce distinct composite signal shapes. **D** Plot of the range of azimuth and inclination values used across experiments. Example composite signals are shown for color-coded circled data points. The azimuth and inclination values were uniformly spaced in the spherical φ and θ space. **E** Timing of each KHF trial. **F** Sample output force recorded from a single KHF trial. Gray boxes indicate force responses used for calculation of stimulation-triggered median force responses. **G** Example stimulus-triggered median where block did not occur (i) and where partial block occurred (ii). The degree of block during the KHF signal was established almost immediately upon starting the KHF signal, with some variability in twitch amplitude during the KHF signal. The 6 s waiting period allowed any onset responses to subside before quantifying the state of block

We quantified responses of the tibial component of the sciatic nerve to KHF signals in anesthetized Sprague-Dawley rats (n = 18 (2 female); 268 to 555 g, median = 400 g; Charles River Laboratories) by recording the force generated by the gastrocnemius (Fig. 1A). All procedures were approved by the Institute for Animal Care and Use Committee of Duke University (Durham, NC) and were in accordance with the Guide for Care and Use of Laboratory Animals (8th edition). The study was also in compliance with the ARRIVE guidelines [10]. The animals were housed under USDA- and AAALAC-compliant conditions, with 12 h/12 h light/dark cycle and free access to food, water, and environmental enrichment. Rats were anesthetized for 2–4 min with 3% isoflurane in air to facilitate subcutaneous injection of 1.2 g/kg urethane. We administered supplemental doses as required (up to 0.5 g/kg total, intraperitoneally). We monitored heart rate and blood oxygenation continuously using a pulse oximeter (PalmSAT 2500 A; Nonin Medical; Plymouth, MN, USA), and we assessed anesthesia depth using the toe pinch reflex and heart rate. We monitored body temperature using a rectal temperature probe (TH-8 Thermalert; Physitemp Instruments, Inc.; Clifton, NJ) and maintained body temperature between ~ 35–38 °C with a heated water blanket. After the experiment, we euthanized rats with 0.5 mL Euthasol intraperitoneally.

We made an incision on the left hind limb from the dorsal midline toward the distal dorsal ankle. We separated the biceps femoris and the vastus lateralis via blunt dissection to expose the sciatic nerve. We cut a portion of the distal biceps femoris to expose the branching of the sciatic nerve and the point of entry of the tibial nerve into the gastrocnemius. We dissected the connective tissue surrounding the sciatic nerve from ~ 0.5 cm caudal to the spinal cord to the location where the sciatic nerve branches into the tibial, common peroneal, sural, and cutaneous nerves. We cut the common peroneal, sural, and cutaneous branches of the sciatic nerve, leaving only the tibial branch intact. We dissected the Achilles tendon, cut its distal end, and tied it to a custom force transducer using umbilical tape. We secured the tibia at its caudal end using a plastic clamp attached to the experimental table.

We placed either a bipolar or a tripolar cuff on the proximal sciatic nerve to deliver test pulses and either a monopolar, bipolar, or tripolar cuff on the distal sciatic nerve (i.e., proximal to the nerve branching point) to deliver the KHF waveforms. We also placed a subcutaneous needle in the right hind limb to serve as a return electrode for stimulation through the monopolar cuff and to measure the impedance of individual contacts, as described below. Each cuff had a 1 mm inner diameter (X-Wide Contact Cuffs, Microprobes; Gaithersburg, MD) and contained one (monopolar), two (bipolar), or three (tripolar) platinum-iridium 90 − 10 ribbon contacts (0.5 mm wide) spaced 1 mm apart edge-to-edge; 1.5 mm of silicone extended beyond the outer edge of each outer contact, and the silicone thickness of all cuffs was 0.7 ± 0.1 mm. After implantation, we measured the distance between the center of the stimulation and blocking electrodes (~ 1 cm) as well as the distance between the center of the blocking electrode and the location at which the tibial nerve entered the gastrocnemius muscle (also ~ 1 cm).

Before implanting each cuff, we measured the impedance of each cuff in saline in its intended configuration (i.e., between the middle contact and shorted outer contacts for tripolar; between the two contacts for bipolar; between the one contact and a needle for monopolar) at 1 kHz only (stimulation cuffs) or at both 1 and 10 kHz (blocking cuffs). We repeated the measurements with the cuffs implanted both at the beginning and at the end of the experiment. In a subset of experiments, we also measured the impedance of each individual contact relative to a needle in the bath (saline) or relative to a contralateral subcutaneous needle (in vivo). Pre-experiment blocking electrode impedances at 10 kHz in vivo were 1.8–3.8 kΩ (median: 2.8 kΩ) for monopolar electrodes, 1–2.3 kΩ (median: 1.9 kΩ) for bipolar electrodes, and 1.3–2.4 kΩ (median: 1.8 kΩ) for tripolar electrodes (A–S1).

We adapted the KHF protocol used in [3] to quantify the responses to KHF signals. We used custom MATLAB scripts to control and synchronize all test pulse, KHF, and recording protocols. An analog voltage signal from a data acquisition unit (NI USB-6216 BNC; National Instruments; Austin, TX) drove an isolated current source (FHC bp Optical Isolator with Probe; Bowdoin, ME; gain set to 0.1 mA/V). The output of the current source was filtered through a circuit identical to that included in [3, 4] to remove DC offsets based on [11]: 1 µF series capacitors along the positive and negative pathways, a 100 kΩ resistor in parallel with the stimulator, and a 100 kΩ resistor in parallel with the load. The current was delivered through a 1 kΩ resistor in series with the stimulation cuff to monitor test pulse shape and amplitude. We delivered symmetric bipolar rectangular test pulses through the stimulation cuff (200 µs pulse width per phase, cathodic phase first on the caudal contact (bipolar) or middle contact (tripolar); 1.45 Hz pulse rate) at several amplitudes from ~ 0.1 mA to 0.9 mA in a randomized order. We selected a stimulation amplitude to use for the test pulses in the KHF trials that was supra-maximal, i.e., an amplitude 0.1–0.2 mA above the minimum required to produce muscle force twitches with a maximal rectified area under the curve of a stimulus-triggered median of three test pulses.

We used MATLAB and a National Instruments VISA connection to set the waveform, frequency, amplitude, and duration of the KHF signal generated by a current source (Keithley 6221; Tektronix, Inc; Beaverton, OR). During each trial, the DAQ delivered a signal to trigger the Keithley 6221 to start outputting a KHF signal at the desired timing. The output of the Keithley 6221 was passed through a 100 Ω resistor, and the voltage across this resistor was supplied as input to a voltage-to-current high power stimulus isolator with 1 MHz bandwidth (A-M Systems 4100; A-M Systems; Sequim, WA) on the 10x input gain setting. This setup allowed the A-M Systems 4100 to function as a current buffer, since loads > 100 Ω—such as the 1-3.8 kΩ loads of our in vivo setup—caused substantial output attenuation from the Keithley 6221, especially at higher frequencies and amplitudes. We filtered the output of the A-M Systems 4100 to eliminate any DC offsets using the same RC circuit described above, and we monitored the KHF signal by visualizing the voltage across a 100 Ω resistor in series with the blocking cuff using a battery-powered oscilloscope (Fluke 190 − 062 ScopeMeter Test Tool; Fluke Corporation; Everett, WA, USA). At the end of each experiment, we also used this approach to measure the intended amplitude of the KHF signal versus the actual amplitude for a range of KHF amplitudes and frequencies that were representative of the signals used during the experiment.

We measured the response to test pulses and KHF signals by amplifying, digitizing, and recording force transducer signals. The signals were amplified by a low-noise voltage preamplifier (SR560; Stanford Research Systems; Sunnyvale, CA) and digitized (20 kHz sampling rate) by a PowerLab 4/35 DAQ (ADInstruments Inc.; Colorado Springs, CO) interfaced via LabChart v7.0 (ADInstruments). We also used the PowerLab to record the DAQ test pulse output differentially to enable stimulus-triggered analysis of force responses.

During each KHF trial (Fig. 1E), we applied 16 test pulses via the stimulation cuff at a rate of 1.45 Hz. After the first four test pulses, we applied a KHF signal via the blocking cuff at a given amplitude, frequency, and waveform shape for 8.1 s.

We reduced power line noise in *post hoc* analysis by estimating—and then subtracting—the noise on a period-by-period basis. This method removed high frequency harmonics of the power line noise (in contrast to notch filtering) while preserving the high frequency dynamics of the signal (in contrast to lowpass filtering). The details of this method are described in the Additional file 1: Section “Artifact Removal”, and the performance of this method is illustrated by Additional file 1: Fig. S2.

We quantified the degree of block and the onset response by comparing the force twitches before the KHF signal and at the end of the KHF signal (Fig. 1F). For each trial, we calculated the stimulus-triggered median of the twitches in response to the first three test pulses (baseline) and the last three test pulses (end of KHF signal) by aligning the three pulses in time and taking the median at every time step (Fig. 1F, G). Thus, any onset response that occurred during the first 6 s of the KHF signal did not affect the assessment of block, although in the absence of onset response, block or partial block occurred almost immediately after the start of KHF. For each stimulus-triggered median signal, we subtracted the mean (i.e., offset) of the pre-stimulus baseline signal. We then calculated the strength of stimulus-triggered responses from the stimulus-triggered median in two separate ways: *AUC*: the area under the curve (using MATLAB’s trapz function) of the rectified stimulus-triggered median; or *max*: the max value of the stimulus-triggered median.

We fit a sigmoid to quantify the degree of block versus KHF amplitude. We defined the degree of block as the ratio of stimulus-triggered response strength (AUC or max values) before vs. during KHF (Fig. 1F, G). A ratio close to zero reflected complete block, while a ratio close to or greater than 1 reflected little to no block. Tonic nerve excitation produced large stimulus-triggered responses that caused this ratio to be much greater than 1. First, we set to 1 all ratios that were larger than 1 to facilitate fitting sigmoidal functions to the data. We then used a nonlinear least squares fit to define a sigmoid for each waveform, each frequency, and each nerve:

1 $$\frac{{metric}_{KHF}}{{metric}_{preKHF}}=\frac{1}{1+\text{exp}\left(-{\upzeta }*(-{I}_{KHF}-\eta )\right)}$$

where *metric*_*preKHF*_ and *metric*_*KHF*_ are the metrics (either AUC or max, as described above) before and during KHF, respectively, *I*_*KHF*_ is the peak amplitude of the KHF signal for a given trial, and *ζ* and *η* are the parameters of the sigmoid fit. We defined ‘block threshold’ as the power, rms, or peak current—with corresponding units of mW, mA_RMS_, or mA—at which 50% ‘partial block’ occurred (i.e., *metric*_*preKHF*_/*metric*_*KHF*_ = 0.5), labelled “*thresh50*”. We calculated ‘block threshold power’ (i.e., thresh50 (mW)) as:

2 $$P=\frac{1}{2}{I}_{max}^{2}{R}_{10 kHz}$$

where *R*_*10kHz*_ was the nerve-specific electrode impedance at 10 kHz, and *I*_*max*_ was thresh50 (mA). Meanwhile, for composite signal tests, we calculated ‘block threshold rms’ (i.e., thresh50 (mA_RMS_)) as the rms of the waveform at a given ‘block threshold current’.

To ensure that we acquired responses to a sufficient number and range of amplitudes to quantify block threshold, we performed a sigmoid fit to AUC data in real time across amplitudes for a given frequency and waveform shape of the KHF signal. We measured responses at five or more amplitudes such that sampling continued until we obtained at least one trial without block (i.e., sub-block threshold amplitude), one to two trials with partial block, and one trial with complete block if block was possible. Further, to facilitate fast sampling of these amplitudes, we automatically generated a list of initial ‘smart amplitudes’ based on previous experiments. We applied these initial amplitudes in a randomized order and then evaluated additional amplitudes as required. In many cases, we stopped sampling when it became clear that a sigmoid fit was inadequate to describe the response to the KHF signal, such as in the absence of block or in the presence of excitation at supra-block threshold amplitude (i.e., re-excitation [12]).

We calculated onset response as the area under the curve of the force recording during the entire KHF signal. In this study, we considered onset response to be the excitation that occurs at the start of a KHF signal that ultimately achieves partial or full block. Thus, our analyses focused on responses to KHF amplitudes that were equal to or greater than block threshold defined by Eq. 1. Given that onset response depends on KHF amplitude, we compared onset response across frequencies, nerves, and waveform shapes by normalizing KHF amplitudes to the block threshold current; we then interpolated onset response at 1.25 or 1.1 times block threshold current. For the relatively small proportion of tests in which the interpolation amplitude was above the range of tested amplitudes, we extrapolated the onset response to be equal to onset response at the highest amplitude tested (i.e., nearest neighbor extrapolation). We used the area under the curve to quantify onset response as opposed to peak force [13] because peak force does not differentiate quickly-decaying onset responses from long, persistent onset responses. We also used area under the curve instead of onset response duration because the duration does not capture onset response amplitude and because our experimental design fixed the duration of the delivered KHF signal.

### Frequency tests

Prior to conducting block threshold measurements, we conducted ‘dummy’ tests for mechanical stabilization of the leg and Achilles-to-force transducer setup; we applied 1, 5, or 10 kHz sinusoids to allow the muscle to contract strongly from excitation at those frequencies. Subsequently, we measured block threshold and onset response for sinusoids at 5, 7, 9, 10, 20, 30, 40, 50, and 60 kHz (Fig. 1B) in a randomized order. For each frequency, we delivered multiple amplitudes to sample the response sigmoid, as described above. We deemed a frequency as ‘non-blocking’ if there was no apparent partial or full block at any of the tested amplitudes. The initial list of randomized amplitudes was informed by data from previous experiments. If none of those initial amplitudes produced block, then we evaluated higher amplitudes. However, to limit exposure to KHF at high amplitudes, we did not increase the amplitude further if [1] responses indicated greater excitation occurring at higher amplitudes or [2] the maximum amplitude tested produced excitation at the current frequency while producing partial or full block at a higher adjacent frequency. We then tested additional integer values of frequencies until we identified a frequency that blocked and an adjacent lower frequency that did not block (with 1–2 kHz precision). We refer to the lowest frequency that blocked as the *minimum blocking frequency*. In *post hoc* analysis, we identified data points manually for which non-blocking frequencies did not meet the above criteria (i.e., in which amplitude sampling was insufficient to determine blocking capability) and excluded those datasets from the analysis of minimum blocking frequency (20,211,123, 20,211,130, 20,211,005). Detailed notes on responses at maximum amplitudes, maximum non-blocking frequencies, and minimum blocking frequencies are included in Additional file 1: Table S1.

We then determined the minimum blocking frequency using three distinct approaches. In Method Manual, we defined the minimum blocking frequency manually as the lowest frequency that blocked within the tested amplitudes after excluding nerves for which the amplitudes were insufficiently sampled according to the criteria described above (Additional file 1: Table S1). In Method AutoAUC and Method AutoMax, we defined the minimum blocking frequency quantitatively as the lowest frequency that had a sigmoid fit with R^2^ greater than 0.5. Method AutoAUC used sigmoid fits based on the area under the curve of stimulus-triggered recordings (i.e., AUC described above), while Method AutoMax used sigmoid fits based on the max value of stimulus-triggered recordings (i.e., peak described above).

We evaluated frequency effects on block threshold and onset response using either a monopolar, bipolar, or tripolar cuff. We fit the block threshold data using two distinct approaches. The first approach fit a quadratic polynomial to block threshold measurements as a function of frequency for each cuff type. We then compared the polynomial fits across cuff types using the mean and 95% confidence intervals of the fits. This approach enabled us to treat frequency as a continuous variable and to characterize the approximate predicted block threshold as a function of frequency for each cuff type. However, this approach assumed that block threshold current or block threshold power followed a quadratic function with respect to frequency. Therefore, we complemented the polynomial fit approach with a linear statistical model that did not assume a quadratic effect of frequency. The linear statistical model treated the frequency as a categorical variable and fit the following mathematical model to the block threshold current and block threshold power data:

3 $$T\left(type,f\right)=\left({c}_{10 kHz}{d}_{bi}\right){d}_{mono}^{{\delta }_{mono,type}}{d}_{tri}^{{\delta }_{tri,type}}{\prod }_{n=6 kHz}^{60 kHz}{c}_{n}^{{\delta }_{fn}}$$

where *T* is the block threshold for a given *type* of cuff (monopolar, bipolar, tripolar) and for a categorical frequency *f* (6, 7, 8, 9, 10, 11, 12, 14, 15, 16, 18, 20, 30, 40, 50, 60 kHz); *c*_*n*_ quantifies the effect of each categorical frequency *n* relative to 10 kHz (e.g., *c*_*6kHz*_ quantifies the effect of 6 kHz relative to 10 kHz); *d*_*mono*_ and *d*_*tri*_ quantify the effect of the monopolar and tripolar cuffs, respectively, relative to the bipolar cuff (*d*_*bi*_), *δ* is a Kronecker delta function (e.g., such that *δ*_*mono,mono*_ = 1 while *δ*_*mono,tri*_ = 0, and *δ*_*10kHz,10 kHz*_ = 1 while *δ*_*10kHz,20 kHz*_ = 0). We defined Eq. 3 such that fit coefficients quantified the effects of electrode geometry and frequency relative to a bipolar cuff (*d*_*bi*_) at 10 kHz (*c*_*10kHz*_). Thus, Eq. 3 coefficients were the scaling factors needed for block threshold of a bipolar cuff at 10 kHz to match the block threshold of the specified cuff type at the specified categorical frequency. We converted Eq. 3 into a linear model via a log (base 2) transformation, enabling us to use standard linear regression to identify the effects of monopolar and tripolar cuffs (*d*_*mono*_ and *d*_*tri*_) relative to bipolar cuffs (*d*_*bi*_) independently of the frequency effects (*c*_*n*_). In cases where we measured block threshold and onset response multiple times for a given frequency, we used the median across replicates for the fits. Polynomial fits used the data from all nerves at all frequencies and all cuff types (n = 194), while Eq. 3 fits used the subset of the data that had measurements for at least two cuff types at any given frequency and at least three distinct nerves at any given frequency (n = 181). We verified approximate normality of residuals using residual histograms, and we report the results of Anderson-Darling tests for normality.

We evaluated interactions between frequency and cuff type by introducing interaction terms to the model in Eq. 3. Since frequency was a categorical variable, the total possible interaction terms between each categorical frequency value and each cuff type could be very large and lead to overfitting. Therefore, we incorporated a single interaction term for each non-bipolar cuff type (monopolar or tripolar) such that the interaction term was active only when categorical frequencies were above a certain cutoff frequency. We identified the appropriate cutoff frequency (14 kHz) empirically by manually sweeping through frequency values from 9 to 29 kHz in 1 kHz increments and selecting the frequency that maximized the adjusted R^2^ of the fit. Since interaction effects with the monopolar cuff were not statistically significant (i.e., the coefficient corresponding to that interaction term was not different from zero), we reduced the number of model terms to include only interactions of the tripolar cuff with frequency. Interaction effects with bipolar cuff were not relevant because the model formulation used the bipolar cuffs as the basis for comparison. Thus, the final model fit with interaction effects was:

4 $$T\left(type,f\right)=\left({c}_{10 kHz}{d}_{bi}\right){d}_{mono}^{{\delta }_{mono,type}}{d}_{tri}^{{\delta }_{tri,type}}{\prod }_{n=6 kHz}^{60 kHz}{c}_{n}^{{\delta }_{fn}}{\prod }_{n=14 kHz}^{60 kHz}{g}^{{\delta }_{fn}}$$

where *g* is the interaction term between the tripolar cuff and frequencies.

### Composite signal tests

We evaluated systematically the effect of waveform shape by generating and testing a set of composite signals, each comprising a sum of two sinusoids (Fig. 1C, D). We constructed a set of waveforms by superposing sinusoids of a given frequency (*f*_*0*_) with sinusoids of twice that frequency (*2f*_*0*_). To vary the shape of the composite signal, we maintained the first harmonic’s phase constant while varying the phase of the second harmonic. We also varied the relative amplitudes of the first and second harmonics. The resulting composite signal was described by:

5 $$s\left( {t,\theta ,\varphi } \right) = cos\left( \theta \right)~cos\left( {2\pi f_{0} t - \pi /2} \right) + sin\left( \theta \right)~cos\left( {2\pi \left( {2f_{0} } \right)t - \varphi } \right),{\text{ }}0{\text{ }} \le {\text{ }}\theta {\text{ }} \le {\text{ 9}}0^\circ ,{\text{ }} - {\text{18}}0^\circ {\text{ }} \le {\text{ }}\varphi {\text{ }} < {\text{ 18}}0^\circ$$

where *s* is the composite signal, *t* is time (in s), *f*_*0*_ is the fundamental frequency (in Hz), *φ—*the *azimuth—*is the phase of the second harmonic, and *θ—*the *inclination—*expresses the relative amplitudes of the harmonics such that a value of 0° produces only the first harmonic (i.e., a single sinusoid with frequency equal to the fundamental frequency, f_0_) while a value of 90° produces only the second harmonic (i.e., a single sinusoid with frequency equal to twice the fundamental frequency, 2f_0_). The range of possible inclination and azimuth values define half of a unit sphere when converting the spherical coordinates *θ* and *φ* into Cartesian coordinates. Thus, we selected 130 unique combinations of inclination and azimuth that sampled this unit half sphere at approximately uniform intervals of surface area. We removed 26 of those combinations that had 90° inclination values (i.e., consisting of only the second harmonic at different phases), leaving 104 combinations of azimuth and inclination values (Fig. 1D) with a maximum inclination value of 78.4°. We evaluated each combination of inclination and azimuth in random order for the first five nerves tested (20,210,908, 20,210,916, 20,211,005, 20,211,007, and 20,211,013). For all subsequent nerves (n = 13), we evaluated azimuth values that were 180° apart consecutively while randomizing the order of these pairs and the order within a pair.

We measured KHF responses to single-frequency sinusoids at f_0_ and 2f_0_ just before starting composite signal tests and after every 12 composite signals. We compared block thresholds and onset responses of composite waveforms to the block thresholds and onset responses for only the first harmonic and of only the second harmonic. We quantified onset response in the same manner described for the frequency tests. However, we quantified block threshold using the rms of the signal at block threshold as opposed to the amplitude of the signal, since the amplitudes of composite signals could depend on the inclination and azimuth values used, while composite signal rms was invariant across inclination and azimuth values.

We evaluated the blocking characteristics of composite signals after running the frequency tests described earlier, and we used the results of those frequency tests to choose nerve-specific fundamental frequencies (f_0_) for the composite signals. We aimed to use relatively low fundamental frequencies to identify the most energy-efficient blocking waveforms possible. In preliminary experiments, the minimum blocking frequency identified in real time (i.e., by Method Manual) could become incapable of blocking or could produce re-excitation later in the experiment, thus making measurements at that frequency challenging. Therefore, for composite signal tests, we often chose fundamental frequencies that were slightly greater than the minimum blocking frequency (Additional file 1: Fig. S1). We excluded the composite signal measurements from one nerve (20,211,130) due to large amounts of excitation during composite signal trials, preventing measurement of block threshold across many composite signals.

## Results

### Frequency effects across cuff types

We compared the minimum blocking frequency, block threshold current and power, and onset response of KHF sinusoidal signals across frequencies with monopolar, bipolar, and tripolar electrode geometries. We analyzed both block threshold current and block threshold power to account for the effect of cuff impedance across cuff types.

#### Minimum blocking frequency, frequency for minimum power, and frequency for minimum Onset

**Fig. 2:**
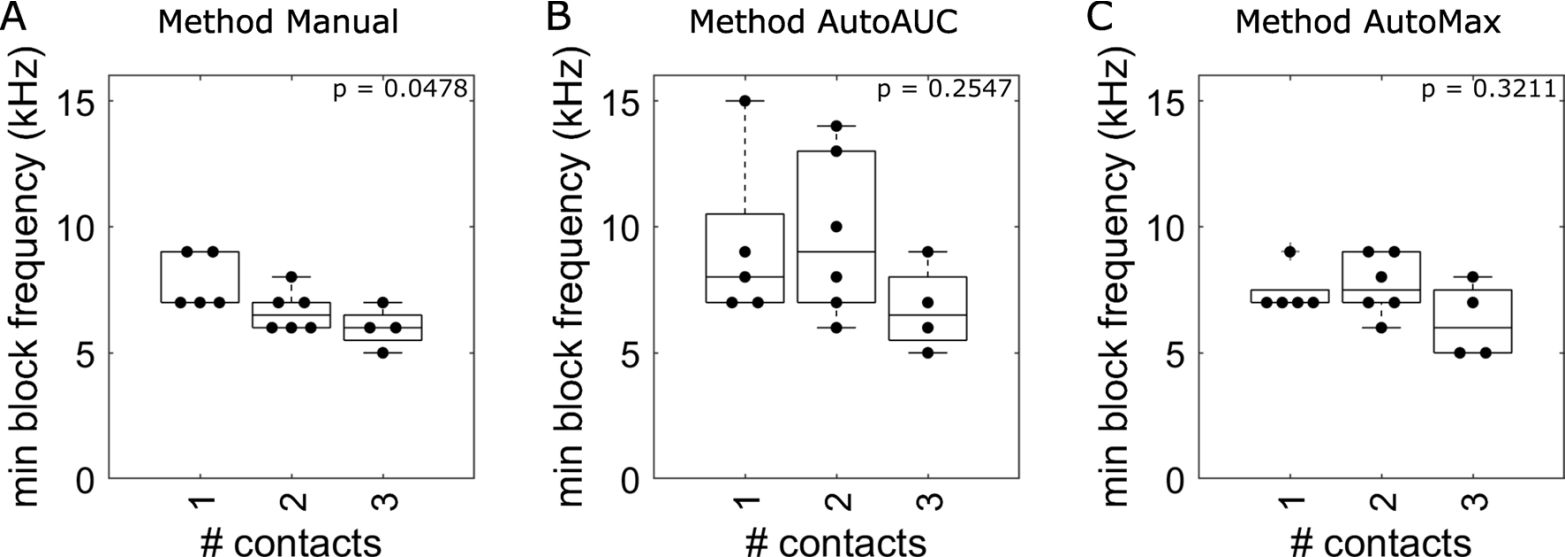
Minimum blocking frequency summary results. Method Manual defined the minimum blocking frequency as the frequency that blocked with a sufficiently large block amplitude window prior to the occurrence of re-excitation (by experimenter observation during data acquisition). Methods AutoAUC and AutoMax defined the minimum blocking frequency as the lowest frequency that had a sigmoid fit with R^2^ greater than 0.5. Method AutoAUC used sigmoid fits based on the *area under the curve* of stimulus-triggered average force. Method AutoMax used sigmoid fits based on the *maximum value* of stimulus-triggered average force. Labeled Kruskal-Wallis test p-values were not corrected for multiple comparisons. Each dot in each panel was a separate experiment on a separate nerve, and no nerve underwent testing with multiple cuffs

Irrespective of the analysis methods, the minimum blocking frequency varied across nerves (Fig. 2), although Method AutoAUC produced a wider range of minimum blocking frequencies (5 to 15 kHz) than Method Manual and Method AutoMax (5 to 9 kHz). While Method Manual indicated a slight difference between minimum blocking frequencies across cuff types (Fig. 2A), there was no difference across cuff types for Methods AutoAUC or AutoMax (Fig. 2B-C). Thus, the minimum block frequency was not strongly influenced by the cuff geometry. While sigmoid fits effectively characterized a majority of our data (Additional file 1: Fig. S3), using Method AutoMax produced better sigmoid fits than using Method AutoAUC when small amounts of re-excitation occurred (Additional file 1: Fig. S4). Therefore, we used Method AutoMax for all subsequent analyses.

**Fig. 3:**
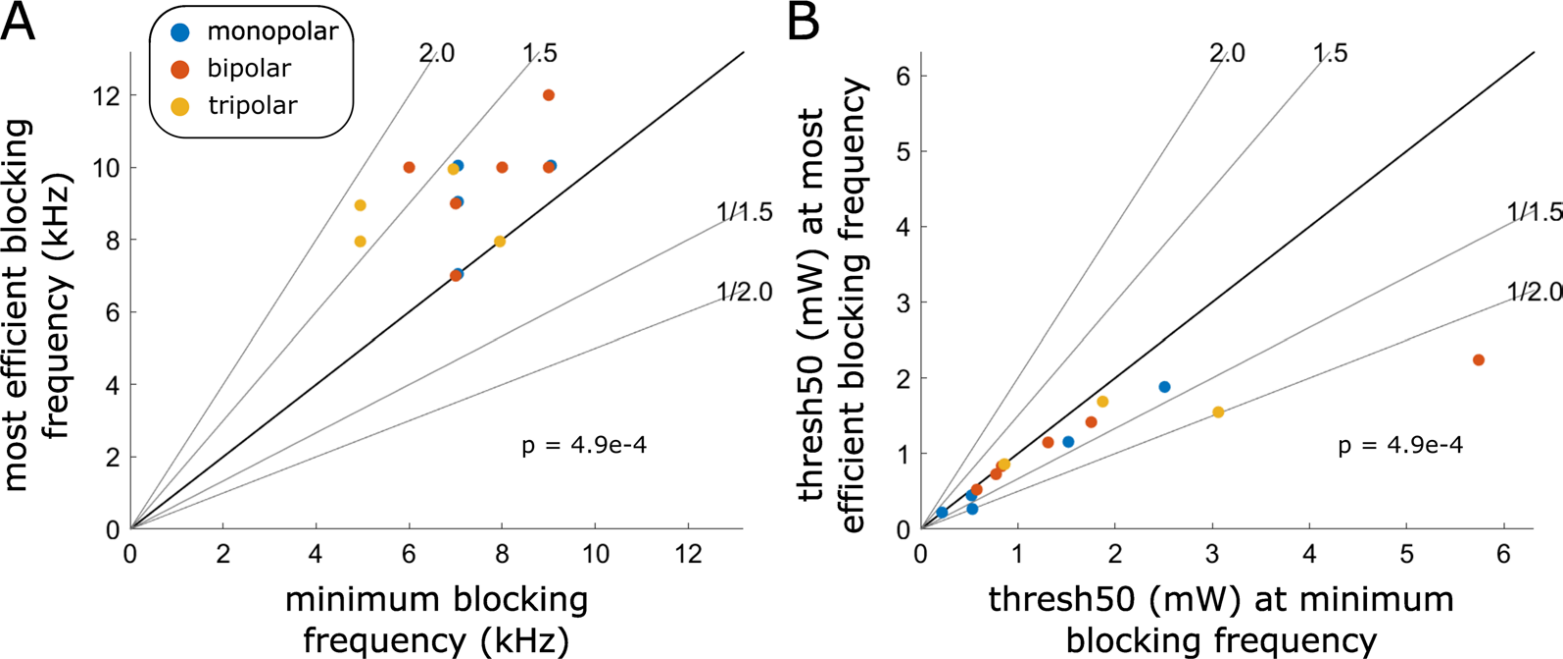
Pairwise comparisons showing that the most efficient blocking frequency occurred at a higher frequency than the minimum block frequency and that it blocked more efficiently than the minimum blocking frequency. **A** Pairwise comparison of data from all nerves and cuff types in terms of frequency. **B** Pairwise comparison of data from all nerves and cuff types in terms of block threshold. No nerve underwent testing with multiple cuffs. For visualization in panel (**A**), both the x-axis and y-axis coordinates of monopolar data points and tripolar data points are offset by − 0.05 Hz (tripolar) or + 0.05 Hz (monopolar). Both panels indicate significantly different pairwise comparisons by a signed rank test at α = 0.05 after multiple comparison correction with Hochberg’s step-up procedure for twelve comparisons (see Additional file 1: Fig. S5 for data from all twelve comparisons)

We compared the frequency, threshold, and onset response for the *most efficient blocking frequency* and for the *minimum blocking frequency*. Compared to the minimum blocking frequency (median: 7 kHz), the most efficient blocking frequency (median: 9 kHz) occurred at a higher frequency (Fig. 3A), had a lower block threshold (Fig. 3B), and had a comparable onset response (Additional file 1: Fig. S5C). This contrasts with the literature on frequency effects [2–5], although additional comparisons aligned with the literature by confirming that even higher frequencies had smaller onset responses and required more energy to block (Additional file 1: Fig. S5D–I). Results were similar when using Method AutoAUC to define minimum blocking frequency (Additional file 1: Fig. S6).

### Frequency effects on block threshold across cuff types

**Fig. 4:**
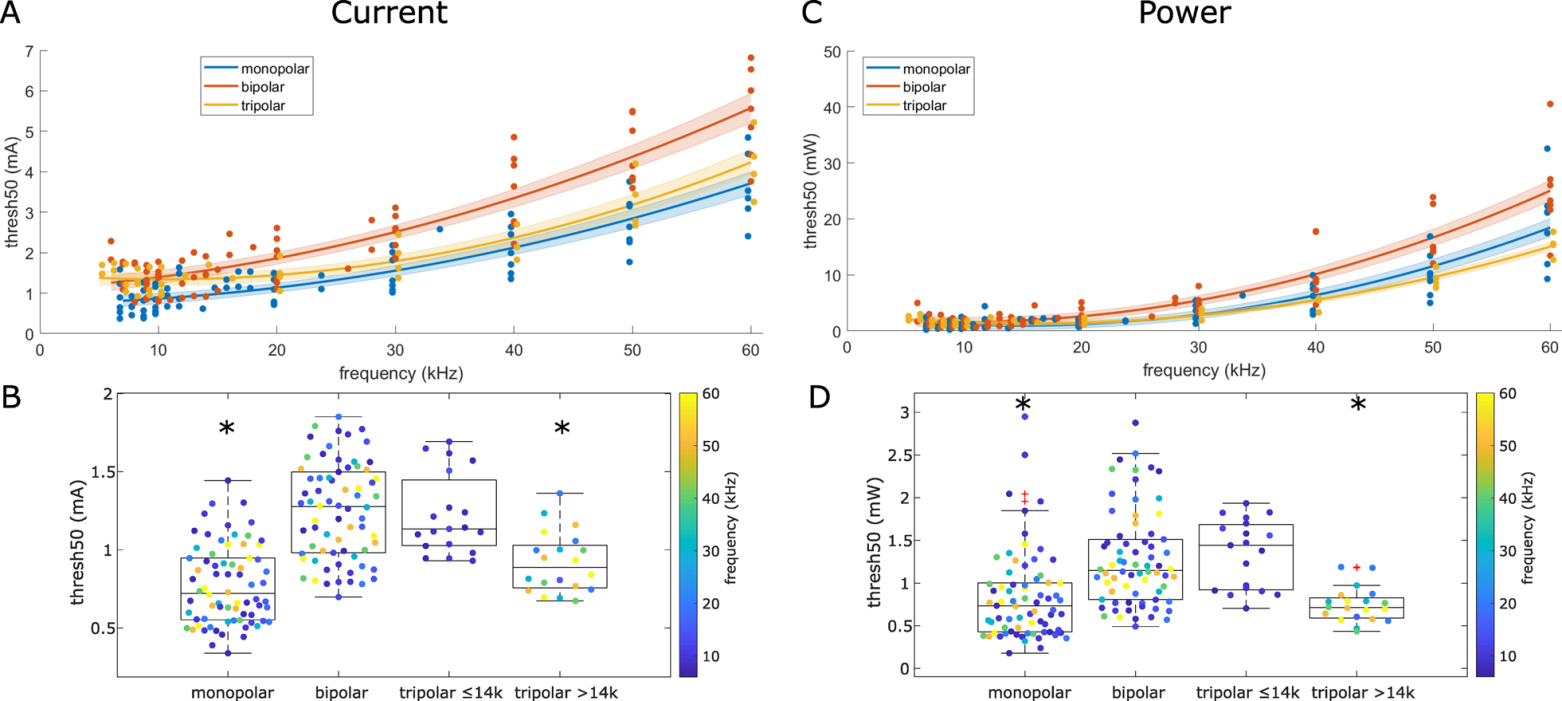
Block thresholds current (left) and power (right) across all nerves, frequencies, and cuff types. **A** and **C** Raw block thresholds and polynomial fits to block thresholds as a function of frequency for each cuff type. Shaded areas show the 95% confidence interval of the fits. See Additional file 1: Table S2 for polynomial coefficients. For visualization, data points are offset on the frequency axis by + 0.25 kHz (tripolar) and − 0.25 kHz (monopolar). **B** and **D** Block thresholds of all measurements (within-nerve replicates averaged as described in Methods) after removing categorical frequency effect (i.e., after dividing thresholds by corresponding *c*_*n*_ coefficients in Eq. 4). Significant differences from bipolar thresholds are denoted by ‘*’. Interaction effects between the tripolar cuff and frequencies greater than 14 kHz are illustrated by plotting the measurements for the tripolar > 14 kHz separately from ≤ 14 kHz. No nerve underwent testing with multiple cuffs

Block threshold current and power increased monotonically as frequency increased from 10 to 60 kHz (Fig. 4A, C). However, this monotonic relationship was no longer clear at frequencies below 10 kHz, and in most cases, the lowest block threshold was not at the minimum blocking frequency (Fig. 3A).

Quadratic polynomial fits (Fig. 4A; Additional file 1: Table S2) and statistical linear model fits (Fig. 4B) revealed that monopolar cuffs had the lowest block threshold current for frequencies below 14 kHz (threshold relative to bipolar cuffs according to Eq. 4 = 0.6083; 95% CI: [0.5510, 0.6715]; p = 2e−18). Tripolar cuffs had similar block threshold currents to bipolar cuffs at frequencies below 14 kHz (threshold relative to bipolar according to Eq. 4 = 0.9598; 95% CI: [0.8149, 1.1305]; p = 0.62) but lower block threshold currents than bipolar cuffs at frequencies greater than 14 kHz (threshold relative to bipolar according to Eq. 4 = 0.7774; 95% CI: [0.6276,0.9630]; p = 0.0214). Monopolar and tripolar cuffs with signals above 14 kHz blocked at comparable currents.

Block threshold power results were analogous to those for block threshold current (Fig. 4C, D): monopolar cuffs had lower threshold power than bipolar cuffs (threshold relative to bipolar according to Eq. 4 = 0.6099; 95% CI: [0.5171,0.7194]; p = 2e−18), tripolar cuffs had similar threshold power as bipolar cuffs below 14 kHz (threshold relative to bipolar according to Eq. 4 = 1.0398; 95% CI: [0.7912,1.3664]; p = 0.78), and tripolar cuffs had lower threshold power than bipolar cuffs above 14 kHz (threshold relative to bipolar according to Eq. 4 = 0.5693; 95% CI: [0.3983,0.8138]; p = 0.0022). Monopolar and tripolar cuffs with signals above 14 kHz blocked at comparable power.

While Anderson-Darling tests indicated that residuals of the linear statistical model fit were only normally distributed for the block threshold power data (p = 0.4135) and not for the block threshold current data (p = 0.0482), the residuals of the linear model were reasonably bell-shaped Additional file 1: Fig. S7) and the confidence interval effect size was sufficiently large that the results are valid.

### Frequency effects on onset response across cuff types

**Fig. 5:**
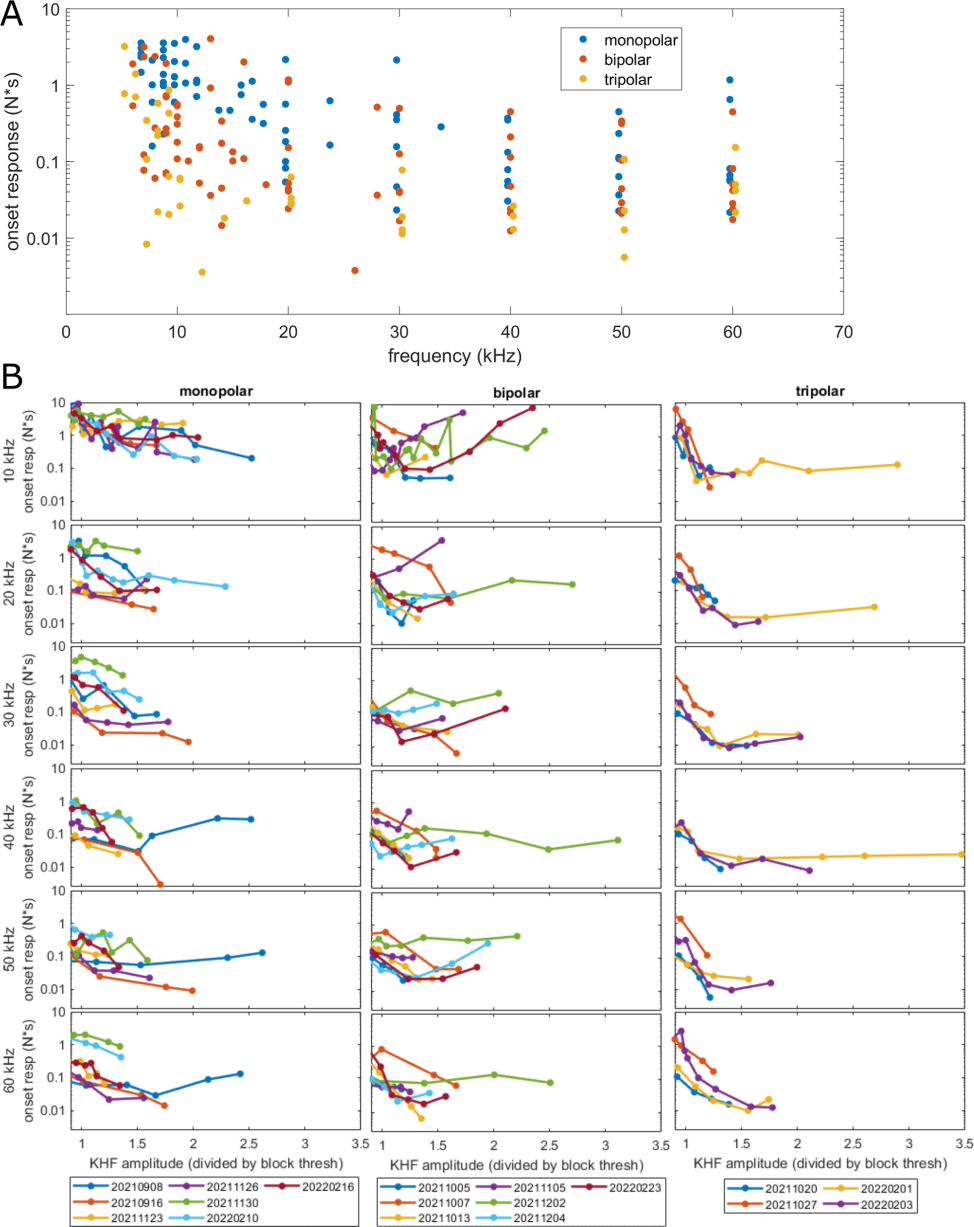
Onset response across frequencies, cuff types, and amplitudes. **A** Onset responses across cuff types interpolated at 1.25 times the block threshold amplitude. For visualization, data points are offset on the frequency axis by + 0.25 kHz (tripolar) and − 0.25 kHz (monopolar). **B** Raw onset responses across all KHF amplitudes. Legend denotes the ID for each nerve. For nerves in which we measured multiple replicates of a given frequency, panel **A** takes the median of the interpolated onset response values, while panel (**B**), which shows all raw data points, only shows the latest of the recorded replicates for visual clarity

Onset response was dependent on frequency, amplitude relative to block threshold, and cuff type. Tripolar cuffs consistently had small onset responses at frequencies of 10 kHz and above. At 1.25 times block threshold, tripolar cuffs produced onset responses < 0.2 N*s at frequencies ≥ 10 kHz, and most monopolar and bipolar cuffs produced onset responses < 3 N*s at frequencies ≥ 20 kHz (Fig. 5A). Onset responses across other amplitudes that were above block threshold showed similar effects of frequency and cuff type (Fig. 5B). Monopolar and bipolar cuffs exhibited a wide range of onset response magnitudes at all frequencies and particularly at 10 and 20 kHz, while tripolar cuffs maintained relatively low onset responses across all frequencies and supra-block threshold amplitudes. Several nerves with monopolar or bipolar cuffs maintained large onset responses at amplitudes that were 1.5 to 3.7 times the block threshold.

### Block with composite signals across cuff types

We constructed a set of periodic waveforms by superposing two sinusoids of differing amplitudes and frequencies: one at *f*_*0*_ and one at *2f*_*0*_ and with given phase. Since our prior work suggested that KHF signals are subject to filtering by axonal membranes [4, 14], the *first harmonic* at f_0_ and the *second harmonic* at 2f_0_ are likely the most influential components of an arbitrary periodic signal during KHF nerve block. This approach of using periodic *composite signals* enables systematic evaluation of the effects of waveform shape on KHF response. We compared the block threshold power and onset response of composite signals across monopolar, bipolar, and tripolar electrode geometries and across a range of inclination and azimuth values.

### Block threshold and onset response of composite signals

**Fig. 6:**
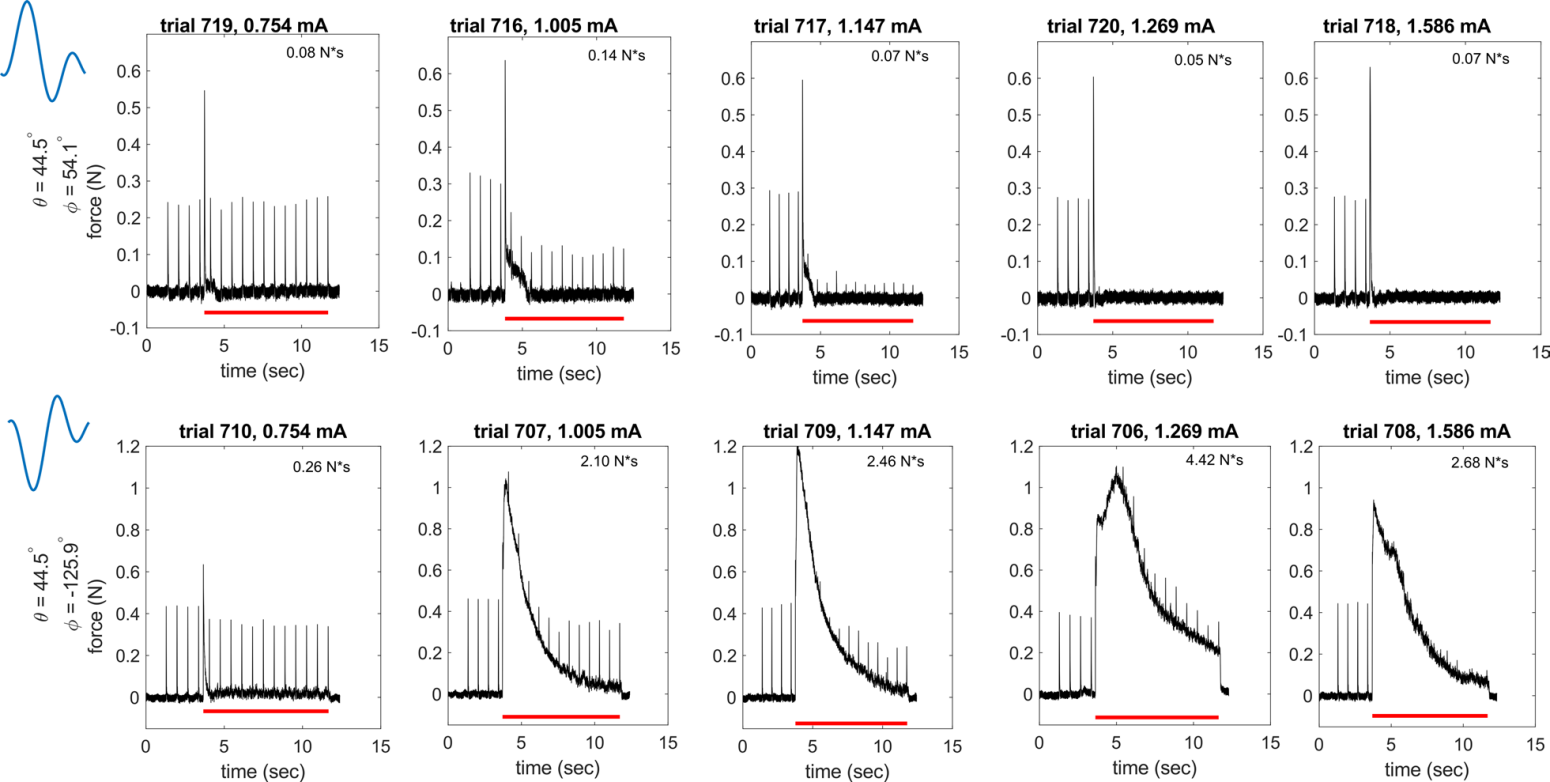
Example of KHF responses to two composite signals (rows) for the same KHF amplitudes (columns). The waveform shape for each row is shown on the left in blue. The top row shows data for azimuth = 54.1°, and the bottom row shows data for azimuth = − 125.9°; thus, the KHF signals are identical except for a 180^o^ phase shift in the second harmonic. The second waveform shape is plotted with a time shift equal to half the period of the signal. Red lines under the data indicate when the KHF signal was on. Onset responses (text in top right corner of each panel) were quantified in N*s. Example is from nerve 20,211,123 (monopolar)

The block characteristics of the composite waveforms depended on the phase of the second harmonic component (i.e., the azimuth value). This was surprising given that the current understanding of block mechanisms does not depend on the phase of the KHF signal or the phase of its constituent elements [4]. As shown in the example of Fig. 6, the waveform with azimuth 54.1° blocked at lower amplitudes with lower onset response than the waveform with azimuth − 125.9°, i.e., a waveform with identical constituent harmonics except for a 180^o^ phase shift of the second harmonic.

**Fig. 7:**
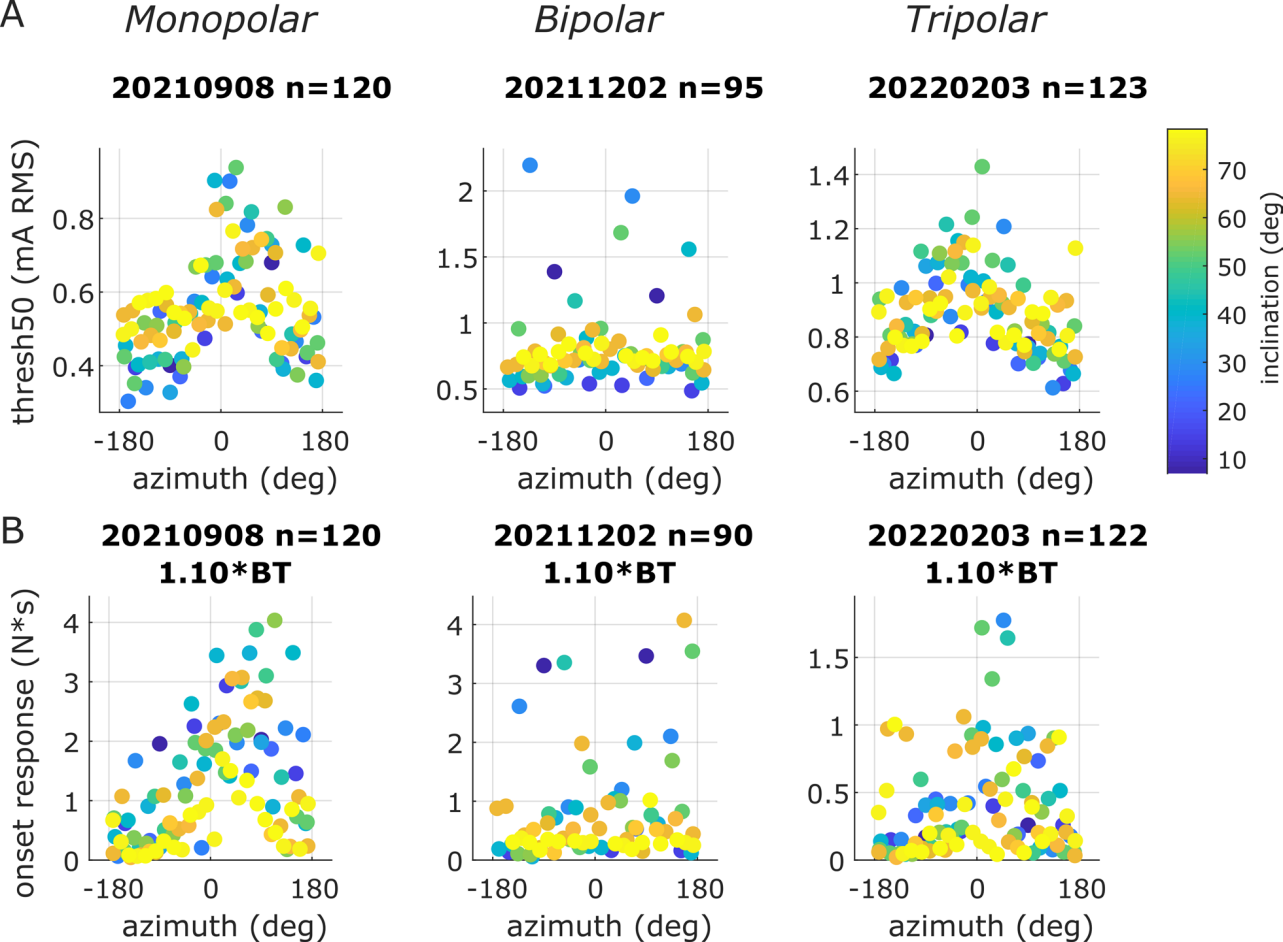
Examples of KHF responses from three nerves (one of each cuff type) in terms of block threshold **A** and onset response **B** vs. azimuth (x axis) vs. inclination (color). Composite signals for all three nerves shown had a fundamental frequency of 10 kHz

The effect of azimuth—at certain inclinations—on block threshold was clearer with monopolar and tripolar electrodes than with bipolar electrodes (Fig. 7A; data for all nerves shown in Additional file 1: Fig. S8A). This effect was present in all monopolar experiments (5 out of 5; Additional file 1: Fig. S8Ai) and in most tripolar experiments (3 out of 4; Additional file 1: Fig. S8Aiii), but there was no such effect in most bipolar experiments (1 out of 7; Additional file 1: Fig. S8Aii). Azimuth values closer to 0° resulted in higher thresholds while azimuth values closer to ± 180° resulted in lower thresholds, indicating that the phase of the second harmonic influenced block threshold.

The effects of waveform shape on onset response were qualitatively similar to the effects on block threshold (Fig. 7B) such that the waveforms (i.e., azimuth-inclination value pairs) with lower block thresholds also had lower onset responses when using a monopolar cuff. This surprising finding suggests that waveform shape could enable a simultaneously improvement in block threshold and onset response, bypassing the previously understood tradeoff whereby lower onset response required a higher frequency—and thus higher threshold—waveform. Onset response depends on the KHF amplitude relative to block threshold; therefore, to isolate the effect of block threshold across waveform shapes, we interpolated onset response at a KHF amplitude that was 1.1 times the waveform-specific block threshold for all waveforms. Waveform shape had a clear effect on onset response at 1.1 times block threshold in all monopolar experiments (5 out of 5; Additional file 1: Fig. S8Bi), but no consistent effect in tripolar experiments (2 out of 4; Additional file 1: Fig. S8Biii) or in most bipolar experiments (1 out of 7; Additional file 1: Fig. S8Bii). In cases where the waveform effect was clear, smaller azimuth values (closer to 0°) had a larger onset response, while azimuth values closer to ± 180^o^ had a smaller onset response; thus, the phase of the composite signal’s 2f_0_ component could determine the size of onset response. For a given azimuth value, higher inclination values (i.e., higher relative proportions of 2f_0_ in the composite signals) resulted in lower onset responses.

### Block threshold and onset response of most efficient and least efficient composite signals

**Fig. 8:**
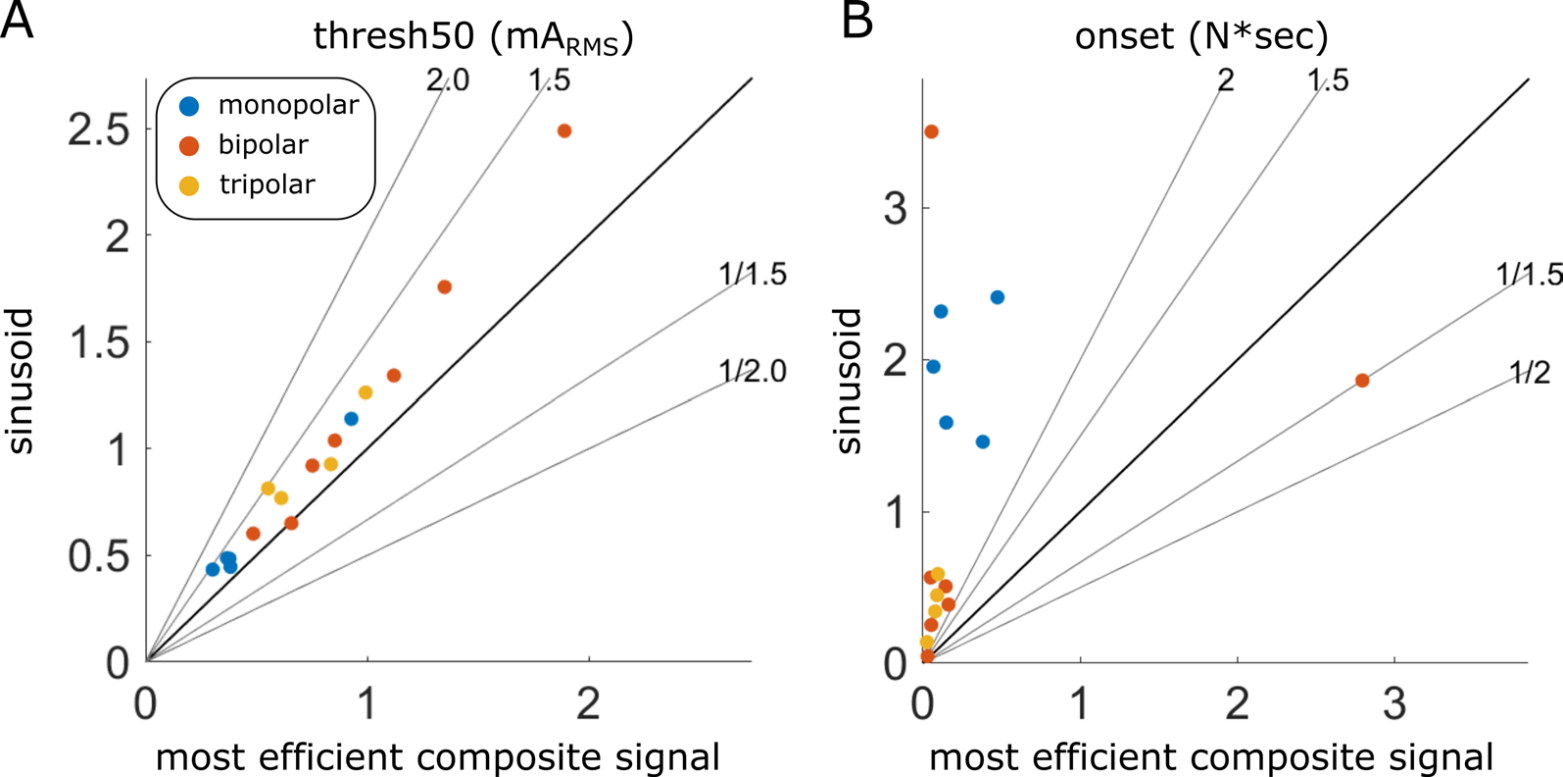
Pairwise comparisons of the most efficient composite signals vs. sinusoids. Sinusoids were at the same frequency—f_0_—as the most efficient composite signal. The most efficient composite signals had lower thresholds and smaller onset responses than sinusoids. Since sinusoids were measured multiple times throughout composite signal tests, the plot shows the median values of those sinusoid replicates. No nerve underwent testing with multiple cuffs

We compared the most and least efficient composite signals to sinusoids. The most efficient composite signals had somewhat lower threshold (0.8x; signed rank test: p = 5.3e−4; Fig. 8A) *and* substantially lower onset response (0.2×; p = 2.7e−3; Fig. 8B) than the sinusoids of the same frequency—f_0_. The most efficient composite signals also had lower threshold (0.6x; p = 4.4e−4; Additional file 1: Fig. S12Di) and comparable onset response (1.2×; p = 0.23; Additional file 1: Fig. S12Dii) as sinusoids of double that frequency—2f_0_. In contrast, the least efficient composite signals had higher threshold (2×; p = 4.4e−4; Additional file 1: Fig. S12Ci) and similar onset response (1.3×; p = 0.20; Additional file 1: Fig. S12Cii) as sinusoids of the same frequency, as well as higher threshold (1.4×; p = 4.4e−4; Additional file 1: Fig. S12Ei) and higher onset response (16×; p = 4.4e−4; Additional file 1: Fig. S12Eii) compared to sinusoids of double that frequency. There were generally no clear interactions between cuff type and the effects of composite signals on block threshold or onset response, although the trends toward lower threshold and higher onset response of monopolar cuffs and lower onset response of tripolar cuffs were consistent with observations from frequency tests. Additional comparisons across different combinations of composite signals and sinusoids are shown in Supplementary Fig. 12.

**Fig. 9:**
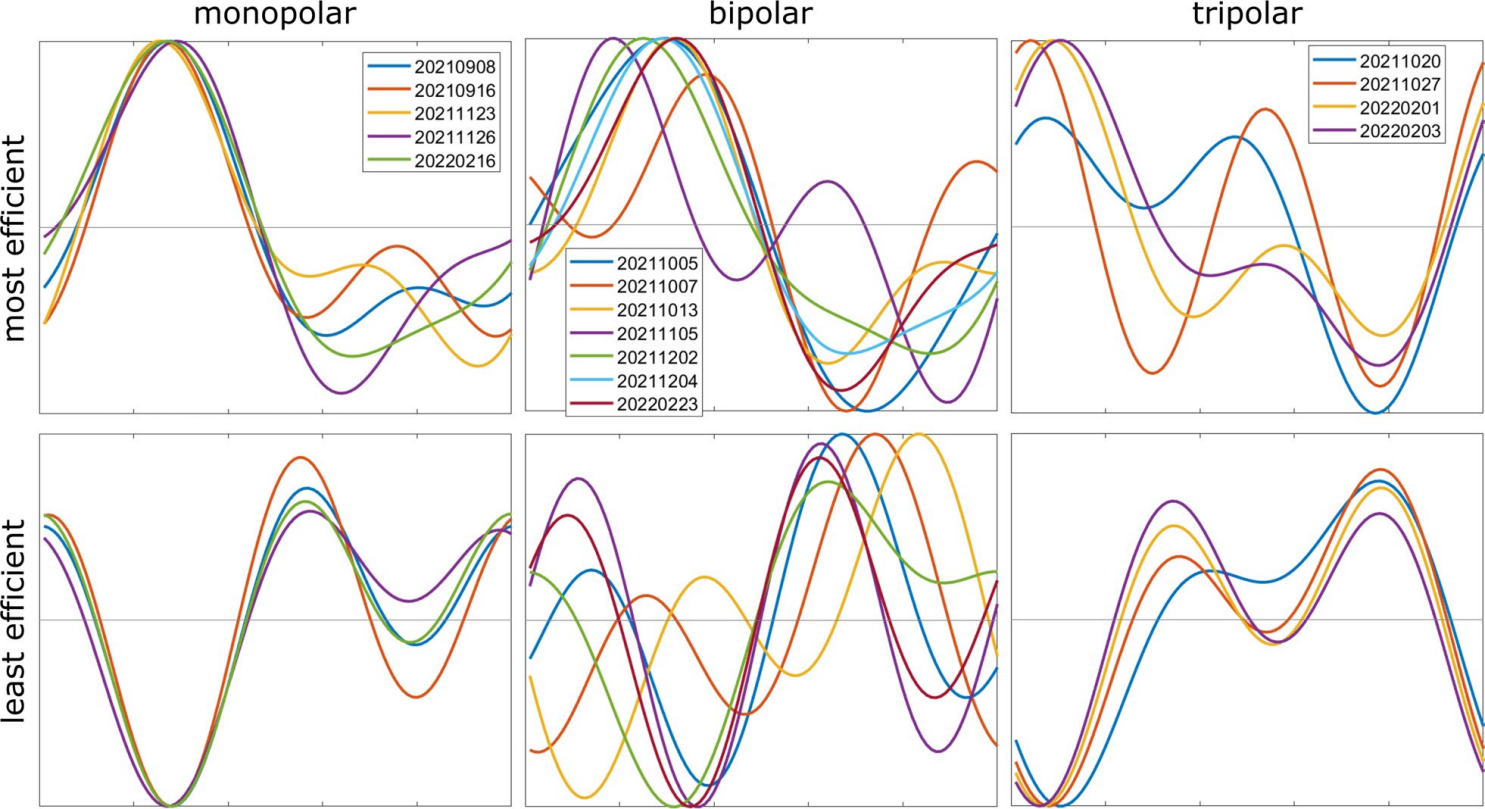
Most and least efficient composite signals across all nerves and cuffs. Efficiency was measured in terms block threshold rms. For visualization, one of the most efficient waveforms for each cuff type (20,210,908, 20,211,005, and 20,211,020) was designated as reference waveforms for that cuff type, and all the most efficient waveforms and the sign-flipped least efficient waveforms were aligned to those reference waveforms to maximize cross-correlation

The most efficient and least efficient composite signals across all nerves are shown in Fig. 9. Composite signal shapes that blocked most efficiently differed across nerves, although some similarities were observed. With monopolar cuffs, the most efficient composite signals tended to have a more prominent positive peak compared to the negative peak, while the least efficient composite signals tended to have a more prominent negative peak. With bipolar cuffs, the most efficient waveforms in 3/7 nerves had stronger positive peaks, but in those cases this feature did not clearly contrast with the least efficient waveforms. With tripolar cuffs, the least efficient composite signals tended to have a stronger negative peak compared to the positive peaks, and the best composite signals in 2/4 nerves had a stronger positive peak compared to negative peaks. With both monopolar and tripolar cuffs, the least efficient waveforms resembled each other across nerves and across cuffs in terms of the prominent negative peak. The most and least efficient waveform shapes in these cuffs also appeared to be opposite polarity versions of each other, although shape differences beyond polarity were also present. Analyses of the peak prominence of waveforms or primary phase duration showed no clear differences between across cuff types (Additional file 1: Fig. S13).

## Discussion

We used concepts from Fourier analysis to generate a set of composite signals that enabled us to probe systematically the effect of waveform shape on KHF nerve block. Varying the waveform shape modulated both block threshold and onset response compared to sinusoids alone. These findings have important implications for the use of waveform design to improve the efficiency and to reduce the onset response of KHF nerve block. Previously, tuning the block threshold or onset response via temporal parameters could only be achieved by increasing the frequency, which is limited by the tradeoff of decreasing onset response at the expense of increasing block threshold. Our present results indicate that composite signals can overcome this limitation by enabling simultaneous reductions in block threshold and onset response. Importantly, these findings were dependent on the electrode geometry: most of the monopolar and tripolar tests with composite signals showed an effect of waveform shape on block threshold and onset response, while most bipolar tests did not. This may explain why previous studies investigating waveform effects—which used only bipolar cuffs—observed waveform effects only on block threshold but not on onset response [4, 6].

### Composite signals

The mechanism by which azimuth (i.e., the phase of the second harmonic) affects block thresholds remains unclear but may be due to the nonlinear response of the membrane to KHF signals. In monopolar and tripolar cuffs, azimuth values close to 0° produced relatively high thresholds and large onset responses, while azimuth values close to 180° produced relatively low thresholds and limited onset responses. Further, a given waveform shape that blocked with high efficiency and low onset response could become markedly less efficient and produce larger onset responses if simply flipped in polarity (i.e., an azimuth change of 180°). While charge imbalances can produce polarity-dependent KHF responses [15], charge imbalances are unlikely to underlie the polarity effects observed here because we used a circuit between the KHF source and the nerve to remove all DC. An alternative explanation is that the axonal membrane responds nonlinearly to the input waveforms. Because composite signals of azimuth values 180° apart contain the same amplitudes of the first and second harmonics, linear filtering must be identical across those waveforms. Therefore, any differences in KHF responses are likely due to nonlinearities of ion channels in the membrane to the timing of waveform dynamics, such that the waveform shape at one azimuth value is more effective at producing and maintaining tonic membrane depolarization than the waveform shape at the opposite azimuth value. Future computational modeling of the membrane response will be helpful to clarify this potential mechanism. Notably, while models do produce a qualitative match of frequency-dependent block thresholds for sinusoids, they underestimate substantially the onset response (i.e., several milliseconds in the models vs. seconds to tens of seconds in vivo) [2, 16], as well as changes therein with waveform parameters. Notwithstanding, the present findings suggest that ‘mixing in’ higher frequency components into a lower frequency carrier alters the KHF response depending on the phase of the higher frequency components.

The present findings reveal a limitation in our previous theory of waveform effects on block thresholds. We proposed that lowpass (linear) filtering of waveforms by the axonal membrane was responsible for frequency and waveform effects on block threshold [4]. We supported this theory by demonstrating that thresholds of non-sinusoidal waveforms were explained by the rms of the signal after lowpass filtering, and that this rms depended largely on the amplitude of the fundamental frequency component resulting from Fourier analysis. This theory explains the observation that square waveforms—with first harmonic amplitude of ~ 1.3 (4/π)—have lower block thresholds than sinusoids [4, 6], as well as the observation that triangular waveforms—with first harmonic amplitude of ~ 0.8 (8/π^2^)—have higher block thresholds compared to sinusoids [6]. However, this theory assumed that lowpass filtering of waveforms was the sole determinant of block threshold, despite the known nonlinear axonal membrane dynamics. In contrast, the present findings showed that composite signals formed from two sinusoids at frequencies f_0_ and 2f_0_ produced KHF responses that depended on the specific temporal dynamics and waveforms shape of the signal (i.e., the phase of the second harmonic relative to the first harmonic) and not simply the constituent frequencies. Our present study leveraged the linear filtering theory to limit the number of harmonics needed to probe KHF responses, and the resulting composite signal methodology provides a technique for characterizing and exploiting the nonlinearity of the axonal response.

The minimum blocking frequency was not the most efficient among the tested frequencies. This finding contradicts prior findings that block threshold increases with frequency [2, 15] and the concept that frequency-dependent block thresholds are due solely to lowpass (linear) filtering by the axonal membrane [4]. One potential explanation is that lower frequencies promote extended activation before producing block at a given amplitude. The dynamics of block reflect a competition between block and excitation that favors excitation when the frequencies and amplitudes are lower. Thus, at lower frequencies, where membrane lowpass filtering plays a limited role, the lower frequency that excites more requires a higher amplitude to generate block. Interestingly, we observed instances of re-excitation in the data at low frequencies (e.g., Additional file 1: Fig. S4﻿). Re-excitation is a phenomenon previously predicted by computational models [12] in which excitation occurs at ‘virtual electrodes’ when applying amplitudes that are sufficiently above block threshold. While previous experimental studies did not show re-excitation, our data indicated that it can occur at low frequencies.

### Cuff effects and cuff interactions with frequency and waveform

Electrode geometry interacted with the effects of waveform. Waveform effects were most prominent with monopolar cuffs, particularly in terms of onset response, while waveform had no effect in most bipolar cuff tests. If each contact on a bipolar cuff had behaved as an independent monopolar cuff, then waveforms that produced block at one polarity but excitation at another polarity—observed in monopolar cuffs—would have likely been evident in bipolar cuffs via the distal contact; the distal contact is closest to the recording site, so the activity evoked at this contact or the block occurring at this contact can drive what is recorded distally. The general absence of waveform polarity effects in most bipolar cuff experiments suggests that the fields and/or responses generated by the two contacts interacted. In contrast, monopolar and tripolar cuff effects were likely dominated by the single contact with the strongest applied current (i.e., the only contact in monopolar cuffs and the middle contact in tripolar cuffs). It is unclear why monopolar cuffs produced relatively larger onset responses and why tripolar cuffs produced relatively smaller onset responses, although the sharper electric field generated by tripolar cuffs may have reduced the number of nodes of Ranvier affected. Given the reduced onset response and the relatively low current/power at block threshold, tripolar cuffs are attractive for efficient and low onset response nerve block. Nevertheless, the relatively simpler geometry of monopolar cuffs and the ability to tune nerve response substantially suggests that monopolar cuffs may be valuable for studying KHF block phenomena to understand the nonlinear dynamics of nodal membranes (e.g., for mechanistic or modeling studies). An important caveat is that results in other nerves with other sizes, morphologies, and fiber compositions may vary. Therefore, evaluating further the effects of KHF temporal parameters (frequency and waveform) in the context of spatial parameters (electrode geometry) remains relevant.

In this study, we evaluated the KHF response of motor fibers (via muscle tension) in the tibial component of the rat sciatic nerve. However, the tibial nerve contains multiple fiber types, which are expected to have different responses to KHF signals. For example, in the rat cervical vagus nerve the thresholds for block differed across fiber types [3], and thus, smaller fibers could be activated by the KHF signal while larger fibers are blocked [17]. Therefore, during the present study we likely only blocked the motor fibers while activating or not affecting smaller fibers. These smaller fiber types are highly relevant for emerging bioelectronic applications to treat various disorders [1, 18, 19], and hence future work evaluating their responses will be important. The effects of frequency, electrode geometry, and waveform shape may be distinct across nerve morphologies and fiber types, and subsequent studies on these fiber types may identify ways to exploit such differences to achieve block selectively.

Frequency effects were similar across cuff types, although the statistical linear models demonstrated an interaction between the spatial and temporal parameters for the tripolar cuff relative to the bipolar cuff. Further, electrode geometry could influence both onset response and block threshold. Block thresholds were highest for bipolar cuffs and lowest for monopolar cuffs. Onset responses were generally smallest using tripolar cuffs and largest using monopolar cuffs, although all cuffs had similarly small onset responses at the highest frequencies. Notably, the tripolar cuff had favorable blocking properties given its relatively lower onset responses and the fact its block threshold current and power were comparable to the monopolar cuff at > 14 kHz. Therefore, when using a single sinusoid for block, a tripolar geometry may be best suited to achieving block in an efficient and low onset response manner.

We considered whether a minimum charge per phase is needed to achieve block irrespective of frequency. Charge per phase at block threshold was relatively constant across frequencies > 20 kHz, but frequencies < 20 kHz required larger charge per phase to block (Additional file 1: Fig. S14), indicating that charge per phase may not account for the observed frequency effects on block thresholds. Our previous work showed that block threshold was predicted better by frequency content of waveforms than by the charge per phase [4]. Further, our computational models showed robust overlap between the membrane’s frequency response and the block threshold vs. frequency effect [4], suggesting a greater role of frequency components than charge per phase.

Our results are consistent with previous findings from rat sciatic nerve showing that onset response tended to increase as the contact spacing of a bipolar cuff increased from 0.5 to 4 mm (i.e., moving toward a monopolar-like geometry) [8]. Meanwhile, our findings that monopolar block thresholds were the lowest contrasted with the previous finding that minimal block threshold occurred at 1 or 2 mm (edge-to-edge) spaced bipolar contacts compared to 0.5 mm or > 2 mm [7]. However, since the electrode geometry in that study differed from ours (e.g., their platinum-iridium ribbons were twice as wide in the longitudinal direction (1 mm vs. 0.5 mm)), it is possible that the optimal inter-contact spacing of our electrodes also differed. Thus, increasing the inter-contact distance to be less than or greater than 1 mm in our cuff geometry may lead to bipolar cuffs having lower block thresholds than monopolar cuffs.

### Minimum blocking frequency

We did not observe block at < 5 kHz in any nerve tested, and most of our minimum blocking frequencies were < 10 kHz. These findings are consistent with previous studies in rat sciatic nerve [20], where block at < 5 kHz was not reported and where 5–10 kHz was reported to either block or produce asynchronous excitation [2, 21]. The minimum frequency for block is understood to vary depending on the system, suggesting a potential role of several stimulation, tissue, and nerve parameters. For example, studies in the frog sciatic nerve [22] and cat pudendal nerve [23, 24] achieved nerve conduction block at frequencies as low as 1 kHz, while others reported needing frequencies of at least 6 kHz in the cat pudendal nerve [25]. Several of the minimum blocking frequencies that we observed using Method AutoAUC were ≥ 10 kHz, which were larger than previous studies in rat sciatic nerve. However, the potential for higher minimum blocking frequencies in certain systems has been reported, e.g., > 20 kHz for the median nerve of non-human primates [26].

While previous studies investigating block threshold across frequency at frequencies as low as 1 kHz reported increasing block thresholds at the lowest frequencies [23, 24], these reports were based on regression analyses that smoothed out the variability in thresholds present at the lowest frequencies. Indeed, linear regression of our own frequency test data would indicate a strong positive correlation between frequency and threshold (not shown) despite such a relationship being inconsistent with thresholds at the lowest frequencies tested.

We deemed as ‘non-blocking’ the frequencies for which we did not observe clear block for the amplitudes tested (Additional file 1: Table S1). However, confirming that these measurements captured the true minimum blocking frequency was challenging due to limited sampling of amplitudes. Limiting the number and maximum value of amplitudes maintained a feasible number of test parameters while mitigating loss of signal that could occur from cumulative KHF exposure [3] or from gradual muscle fatigue. While it is possible that increasing the amplitude may have produced block at some of the frequencies deemed as ‘non-blocking’, we found that the highest amplitude tested for non-blocking frequencies produced block at the minimum blocking frequency. Thus, the conclusion that the lowest frequencies are not maximally efficient remains supported by the data.

### Reduced nerve conduction

We observed variability in responses within a nerve across time and between nerves. We selected nerve-specific fundamental frequencies to try to maximize blocking efficiency while still achieving consistent block. Selecting the lowest such frequency also helped mitigate the impact of membrane lowpass filtering that reduces the effect of higher frequencies. Nevertheless, the use of nerve-specific fundamental frequencies likely contributed to the variability in KHF response observed across nerves. In addition to individual differences in nerve size, fascicular structure, and fiber composition, other potential sources of variability were differences in impedance between contacts of a given cuff (Additional file 1: Fig. S1) and the nerve condition over the course of the experiment. The potential for contact asymmetry in bipolar and tripolar cuffs after implant can lead to differences in electric potential distributions along the nerve, which may produce results that approach those of the monopolar cuff tests (for bipolar cuffs) or bipolar cuff tests (for tripolar cuffs). This could occur due to differences in conformation of the cuff-to-nerve interface such as distance to the nerve at each contact, resulting in distinct electric potential distributions even for current-controlled stimuli. Finally, we applied KHF signals and evoked muscle responses for several hours in each experiment, and these procedures resulted in differing degrees of long-term drift in nerve responses (Additional file 1: Figs. S9–S11). Our analyses neglected this drift and long-term effects on the nerve, although the experimental design of a subset of experiments (see Methods) measured response of waveforms with ‘opposing’ azimuth value pairs immediately after one another partially to address this issue. Despite drift, the remarkable consistency of azimuth values that maximized or minimized response variables provides compelling evidence that waveform shape effects exist and are generalizable.

## Conclusions

The minimum blocking frequency was not the most efficient blocking frequency, monopolar cuffs blocked with the lowest current, monopolar and tripolar cuffs blocked with the lowest power, and tripolar cuffs had the lowest onset response. We developed a composite signal method to probe systematically KHF response to waveform shape, and waveform shape could produce markedly different block thresholds and onset responses depending on the relative phase and amplitudes of constituent sinusoids. These waveform effects were dependent on electrode geometry, with monopolar cuffs exhibiting the strongest waveform effects. Taken together, the insights obtained here motivate—and provide new strategies for—future investigations into the role of spatiotemporal parameters for KHF nerve conduction block. These findings also provide new insights for translation of efficient and low onset response KHF nerve block interventions.

## Supplementary Information

**Additional file 1. **Additional tables and figures.

## Data Availability

Delivered stimulation signals (KHF amplitudes, KHF waveforms), analyzed outputs (block thresholds, onset response values), and all associated metadata from all frequency tests and composite signal tests were uploaded to Duke University’s Research Data Repository and are publicly accessible at 10.7924/r4w37z317. The code to produce all the figures is also available at the same repository as the data.

## Abbreviations

KHF: Kilohertz-frequency
AUC: Area under the curve
