# Supplementary material for: Spatiotemporal parameters for energy efficient kilohertz-frequency nerve block with low onset response
Source: J Neuroeng Rehabil. 2023 Jun 5;20:72. doi: 10.1186/s12984-023-01195-8 (PMC10240787; doi:10.1186/s12984-023-01195-8)
Supplement: Supplementary file 1 — Additional file 1. Additional tables and figures. [file 12984_2023_1195_MOESM1_ESM.docx]

Additional file 1


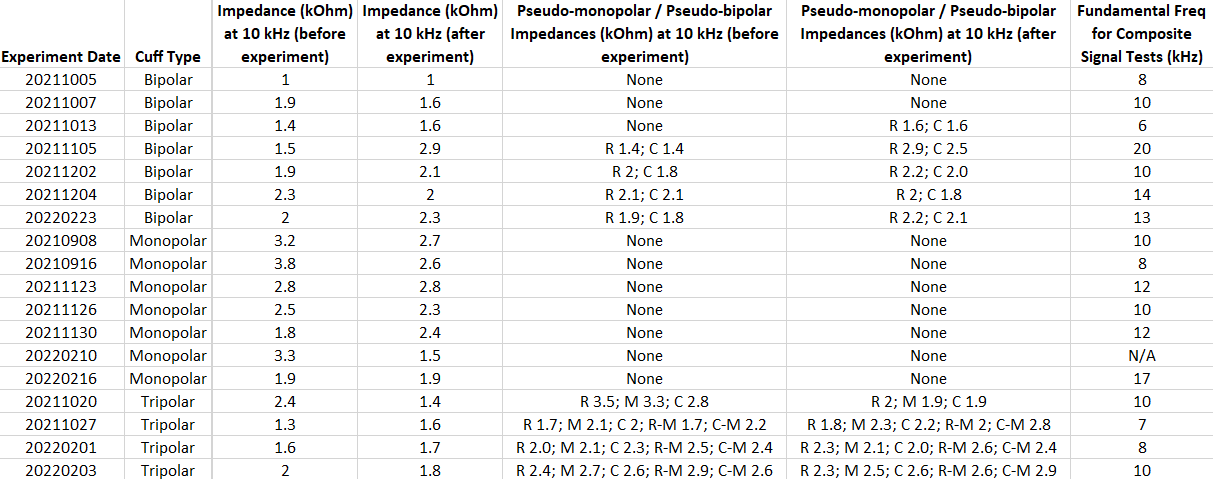


Additional file 1: Figure S1. Nerve identification numbers (“Experiment Date”), cuff types used, impedances, and fundamental frequency used for composite signal tests. Impedances were measured in the configuration used during experiment (third and fourth columns) or as individual contacts relative to either a contralateral subcutaneous needle or one of the other contacts in the cuff (fifth and sixth columns). Contacts in non-monopolar cuffs are referred to as 'R' [rostral], 'M' [middle] (for tripolar), or 'C' [caudal]. Impedances separated by a semicolon refer to the impedance of a given contact referenced to the contralateral subcutaneous needle (e.g., 'R') or of one contact relative to another (e.g., 'R-M'). All impedances shown were measured with the cuff on the nerve.

Additional file 1: Table S1. Maximum non-blocking frequency, minimum blocking frequency, maximum amplitude tested for maximum non-blocking frequency, and additional notes on postdoc manual determinations of minimum blocking frequency.

| **nerve ID** | **cuff type** | **max non-block freq (Hz)** | **max amplitude tested (mA) for max non-block freq** | **min block freq (Hz)** | **notes** |
| --- | --- | --- | --- | --- | --- |
| 20210908 | Monopolar | 6 | 0.859 | 7 | 7 kHz almost blocked fully by 0.62 mA and 0.859 mA, while 6 kHz did not block at all at any of those; also, the largest amplitude tested had larger excitation than lower amplitudes, suggesting that higher amplitudes wouldn't help block |
| 20210916 | Monopolar | 6 | 1 | 7 | 7 kHz almost blocked fully by 0.7 mA and 0.757 mA, while 6 kHz did not block at all at any of those; also increasing 6 kHz to 1 mA caused a mild increase in excitation, suggesting that higher amplitudes would not help |
| 20211005 | Bipolar | 8 | 2 | 10 | exclude this from 'min freq' analysis since the system changed such that later in the experiments 6 kHz blocked and 8 kHz blocked, so min freq unclear |
| 20211007 | Bipolar | 5 | 2.267 | 6 | 6 kHz blocked almost fully by 2.130 mA and 2.267 while 5 kHz didn't block at any of those and had higher excitation response at 2.267 mA than at 1.998 mA suggesting that raising the amplitude wouldn't help |
| 20211013 | Bipolar | 5 | 2.75 | 6 | 6 kHz blocked almost fully by 1.5 mA while 5 kHz didn't clearly block even at various amplitudes at or below or above 1.5 mA |
| 20211020 | Tripolar | 4 | 2.2 | 5 | 5 kHz blocked almost fully by 1.7 mA, while 4 kHz didn't block at any tested amplitudes, and the highest amplitude tested had higher onset response than a lower amplitude, suggesting that raising the amplitude wouldn't help |
| 20211027 | Tripolar | 5 | 2 | 6 | 6 kHz had strong partial block by amplitudes 1.793 and 1.830 mA, while 5 kHz had excitation at every tested amplitude; although onset response at 1.522 mA was slightly higher than at 2.0 mA so perhaps raising further might have blocked |
| 20211105 | Bipolar | 5 | 2.294 | 7 | 7 kHz had strong partial block by 1.3, 1.4, and 1.5 mA, while 5 kHz excited strongly at all amplitudes tested |
| 20211123 | Monopolar | 5 | 0.6 | 7 | exclude this from 'min freq' data since we stopped testing 5 kHz prematurely; there was no indication that 0.6 mA was the appropriate max since 7 kHz didn't start to block until 1.47 mA |
| 20211126 | Monopolar | 6 | 1.25 | 7 | 7 kHz blocked fully by 0.7 mA while 6 kHz only excited at all tested amplitudes, and the highest amplitude tested had higher excitation than a couple of lower amplitudes, suggesting that raising the amplitude wouldn't help |
| 20211130 | Monopolar | 5 | 0.99 | 7 | exclude this from 'min freq' data since we stopped testing 5 kHz prematurely; there was no indication that 0.99 mA was the appropriate max since 7 kHz didn't start to block until 1.8 mA |
| 20211202 | Bipolar | 7 | 1.812 | 8 | 8 kHz blocked fully by 1.054 mA while 7 kHz only excited at all tested amplitudes |
| 20211204 | Bipolar | 5 | 1.6 | 7 | 7 kHz had strong partial block by 1.3 mA while 5 kHz produced strong excitation at all tested amplitudes |
| 20220201 | Tripolar | 5 | 3 | 6 | 6 kHz almost blocked fully by 1.5 mA while 5 kHz produced excitation at all tested amplitudes |
| 20220203 | Tripolar | 6 | 1.842 | 7 | 7 kHz blocked fully by 1.057 mA, while 6 kHz produced excitation at all tested amplitudes |
| 20220210 | Monopolar | 7 | 1.5 | 9 | 9 kHz blocked fully by 1.5 mA while 7 kHz produced excitation at all tested amplitudes |
| 20220216 | Monopolar | 7 | 2 | 9 | 9 kHz blocked fully by 1.4 mA while 7 kHz produced excitation at all tested amplitudes, and 2 mA produced more excitation than 1.5 mA suggesting that raising the amplitude wouldn't help |
| 20220223 | Bipolar | 5 | 1.5 | 6 | 6 kHz blocked fully by 1 mA while 5 kHz produced excitation at all tested amplitudes |

Additional file 1: Table S2. Equations for polynomial fits in Figure 4A and 4C.

| **Cuff type** | **Parameter fit** | **Parameter as a function of frequency, f (kHz)** |
| --- | --- | --- |
| Monopolar | Current Threshold (mA) | 0.0007489(f-20)^2^ + 0.03458(f-20) + 1.134 |
| Bipolar | Current Threshold (mA) | 0.0009236(f-20)^2^ + 0.0559(f-20) + 1.86 |
| Tripolar | Current Threshold (mA) | 0.001182(f-20)^2^ + 0.02238(f-20) + 1.446 |
| Monopolar | Power Threshold (mW) | 0.008615(f-20)^2^ + 0.08794(f-20) + 1.16 |
| Bipolar | Power Threshold (mW) | 0.008978(f-20)^2^ + 0.2002(f-20) + 2.68 |
| Tripolar | Power Threshold (mW) | 0.006736(f-20)^2^ + 0.06335(f-20) + 1.431 |

## Artifact Removal

We removed line noise (i.e., of nominal frequency 60 Hz) by subtracting from the signal the estimated power line artifact at each artifact window (i.e., at each window of nominal duration 0.017 s). We estimated the artifact at each artifact window as the median of all the artifact windows within 0.25 seconds of the current window. To prevent low-frequency signal components of interest from being subtracted with this method, we estimated the artifact from a highpass filtered version of the signal (>54 Hz). Since the period of the artifact differed slightly from the nominal value of 1/(60 Hz), the artifact had a different phase across adjacent artifact windows, resulting in less effective artifact estimation when taking the median across adjacent artifact windows. Therefore, we used FFT to determine the frequency of the power line artifact precisely with 0.001 Hz precision (e.g., 60.017 Hz). To facilitate taking the median across windows within 0.25 seconds, we also resampled the signal (MATLAB’s interpft) such that each artifact window had the same number of data points as every other artifact window. Finally, we applied a moving mean (MATLAB’s movmean) window of 0.5 ms to reduce spurious spikes that were not removed by the period-based artifact removal above. This filtering method enabled substantial reduction in the non-sinusoidal powerline noise artifacts (Supplementary Figure 2) while preserving high frequency dynamics of muscle twitches. This artifact removal code will be made publicly available.


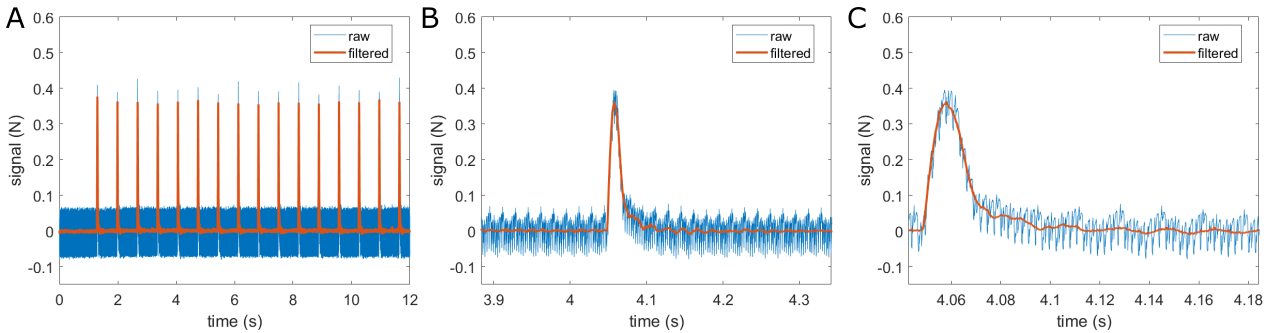


Additional file 1: Figure S2. Example of power line noise reduction. (A) Full trial view. Peak twitch amplitudes were more variable in the raw data than in the filtered data, indicating that the extra variability was due to superposed power line noise. (B) Zoomed in view on a single twitch. Filtered data preserved high frequency dynamics of force recordings. Small ripples after the twitch had a frequency of 53 Hz. (C) Further zoomed in view on a single twitch. The power line noise artifact was periodic and non-sinusoidal. Filtering reduced the artifact substantially.


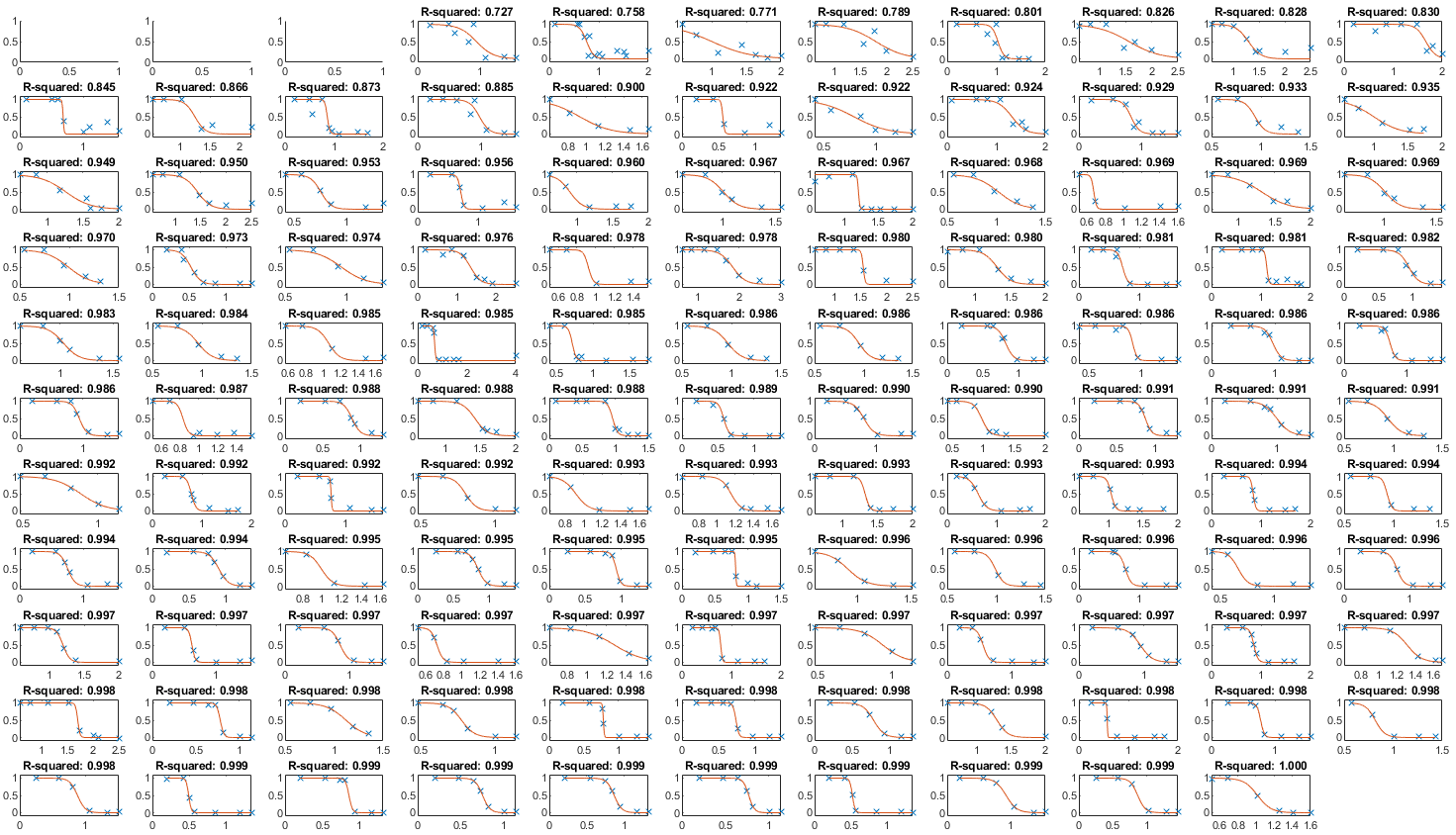


Additional file 1: Figure S3. Examples from all the composite signal tests for nerve 20210908 showing that the sigmoid fits using Method AutoMax provided a very good characterization of the collected data. Sigmoid fits are sorted (from left-to-right and top-to-bottom) by R-squared value. The empty subplots had AUC values greater than 0.25 for all amplitudes, and thus had no valid block threshold value.


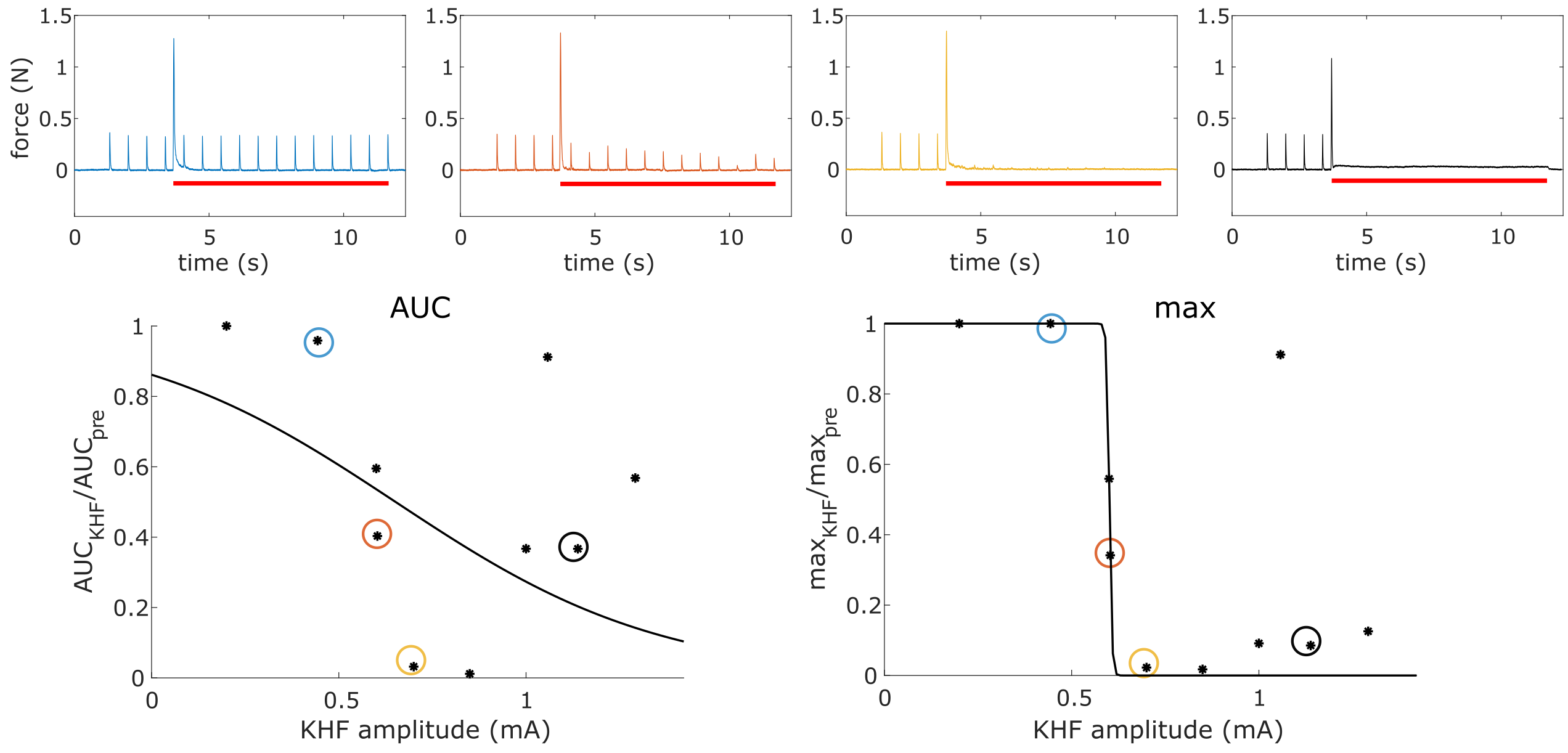


Additional file 1: Figure S4. Example showing that Method AutoMax produced better sigmoid fits than Method AutoAUC in the presence of mild re-excitation. The proportion of data with such re-excitation was small. Raw traces of force (in newtons) correspond to color-coded circles in sigmoid fits. Red lines under the raw traces indicate when the KHF signal was on.


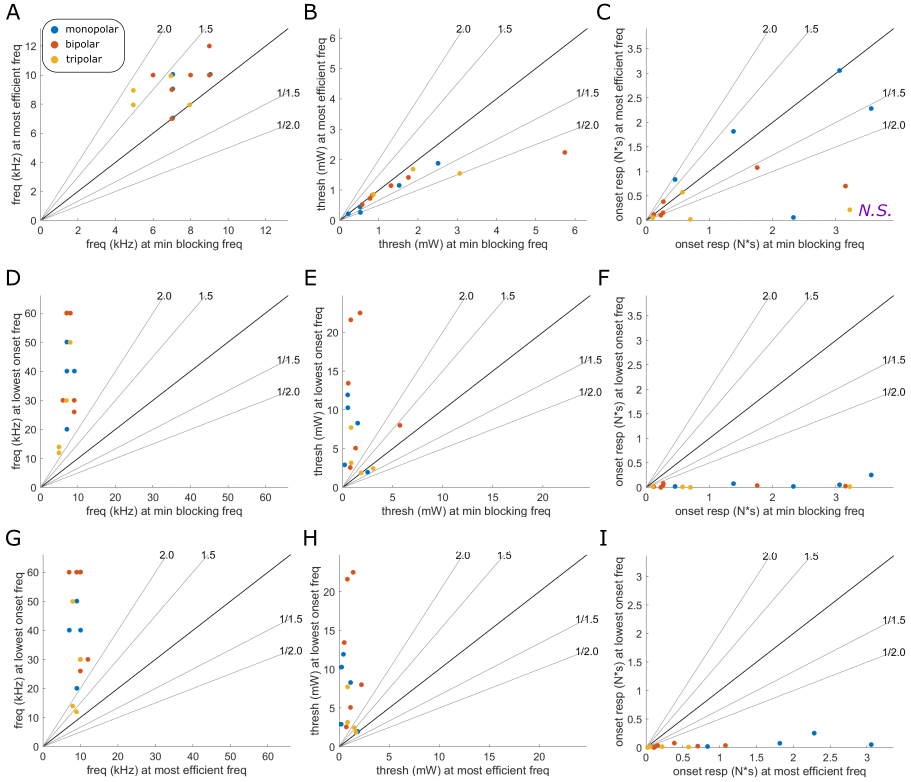


Additional file 1: Figure S5. Pairwise comparisons of frequency (left), block threshold power (in mW) (middle), and onset response (right). The two comparison types were: (top) at the most efficient blocking frequency vs. at the minimum blocking frequency; (bottom) at lowest onset response frequency vs. minimum blocking frequency. No nerve underwent testing with multiple cuffs. For visualization in panels (A) and (D), both the x-axis and y-axis coordinates of monopolar data points and tripolar data points are offset by -0.05 Hz (tripolar) or +0.05 Hz (monopolar). All panels except the one with “N.S.” had significantly different pairwise comparisons by a signed rank test at α=0.05 after multiple comparison correction with Hochberg’s step-up procedure for twelve comparisons. Minimum onset response occurred at a higher frequency (12 to 60 kHz; median: 40 kHz) than both the minimum blocking frequency (panel D; 5.000 [2.414, 8.571]; p=6.1e-05) and the most efficient blocking frequency (panel G; 3.985 [1.335, 8.571]; p=6.1e-05). At those higher frequencies, onset response was substantially smaller compared to the minimum blocking frequency (panel F; 0.022 [0.005, 0.294]; p=6.1e-05) and compared to the most efficient blocking frequency (panel I; 0.083 [0.018, 0.358]; p=6.1e-05). Meanwhile, block thresholds that generated the minimum onset response were much larger compared to the minimum blocking frequency (panel E; 5.458 [0.787, 25.909]; p=6.1e-04) and compared to the most efficient blocking frequency (panel H; 7.177 [1.049, 38.579]; p=6.1e-05).


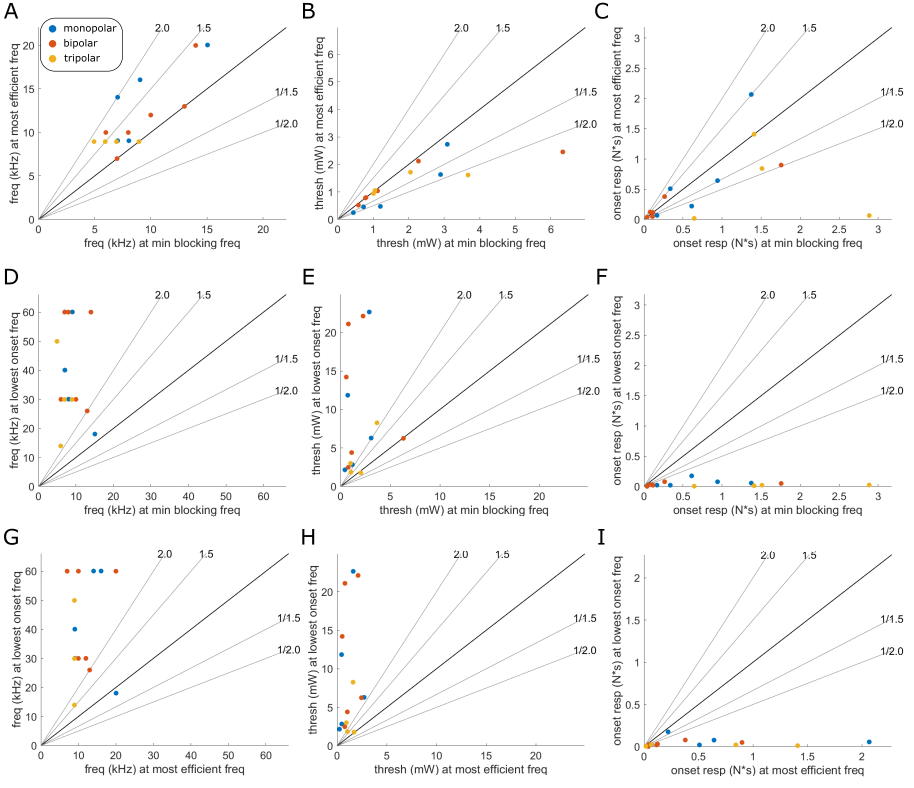


Additional file 1: Figure S6. The same data as in Supplementary Figure 5, but using Method AutoAUC (i.e., AUC) to calculate block thresholds and minimum blocking frequency. Results were similar irrespective of the method used.


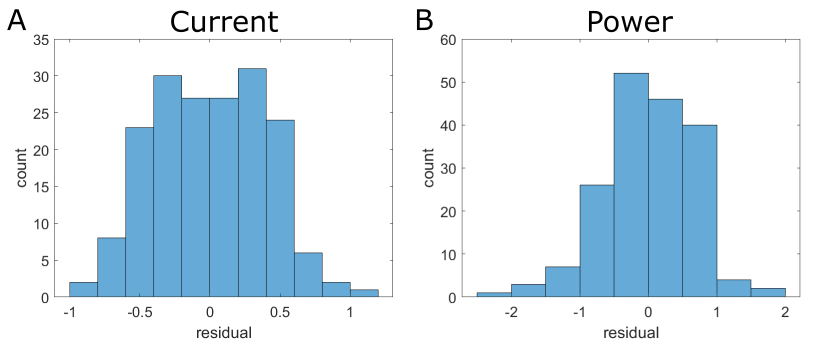


Additional file 1: Figure S7. Residuals of fitting Equation 4 to block threshold current data (A) and block threshold power data (B).


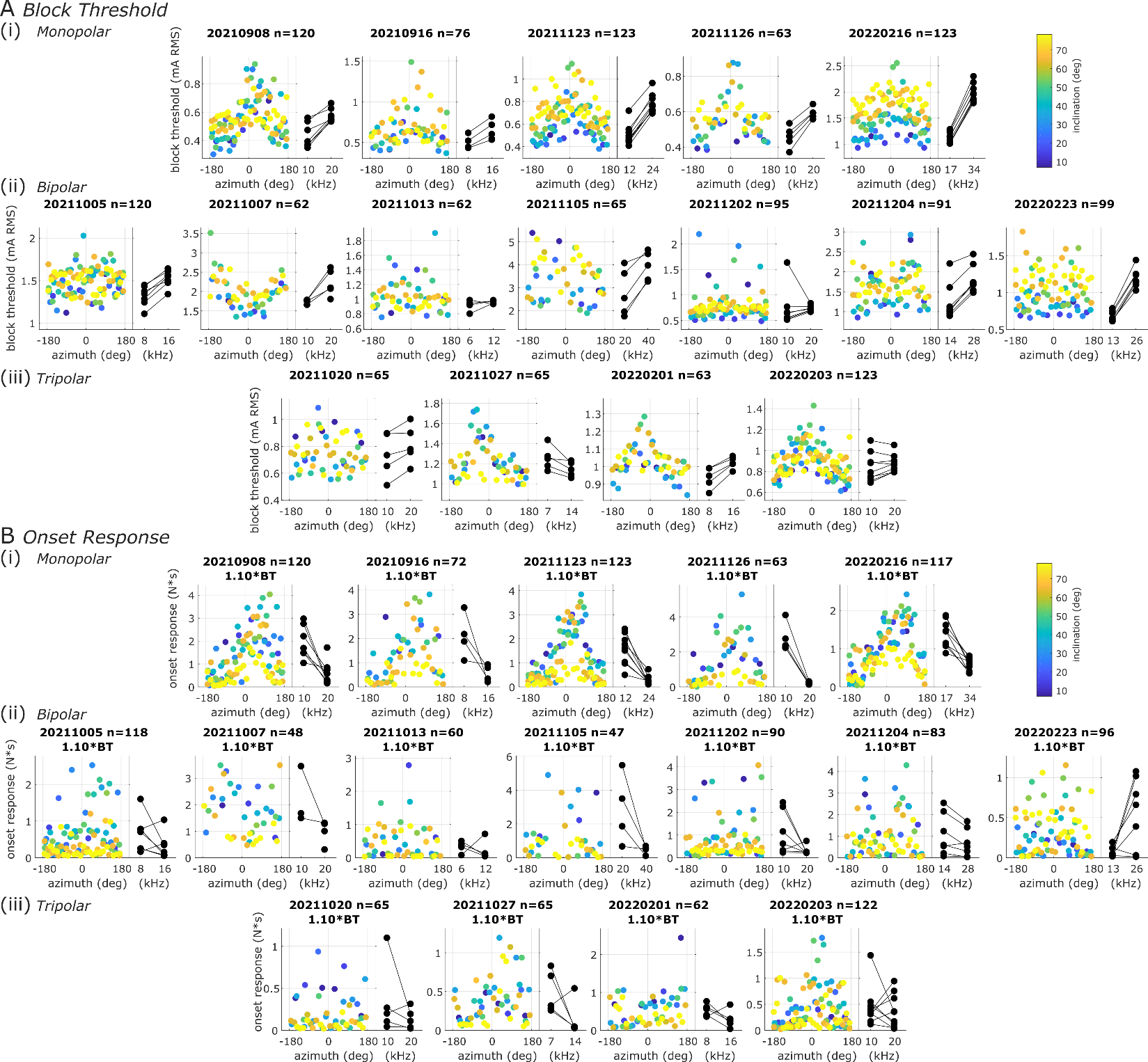


Additional file 1: Figure S8. Block threshold (A) and onset response (B) vs. azimuth (x axis) vs. inclination (color). Corresponding nerve-specific block thresholds for single sinusoids at f_0_ and 2f_0_ are shown in black; black data points connected by a line were measured consecutively. Single sinusoid tests were repeated throughout the composite signal tests after every 12^th^ composite signal measurement. Each colored and black data point in a given panel is from a single block threshold measurement. No nerves underwent testing with multiple cuff types. The number of data points differed across nerves (range: 63-123) due to a loss of signal from muscle fatigue and/or cumulative nerve conduction loss from long-term application of KHF.


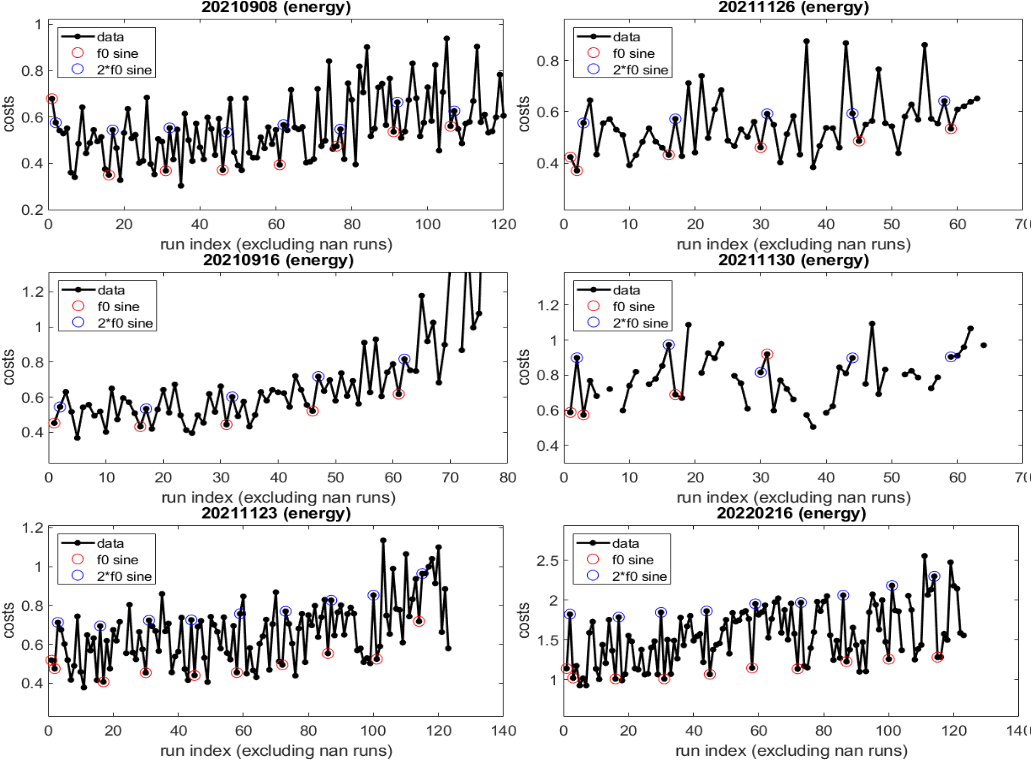


Additional file 1: Figure S9. Block thresholds in mA rms (“cost”) for all composite signal tests in monopolar cuffs. The circled data points were single sinusoids at the fundamental frequency (red) or at twice the fundamental frequency (blue).


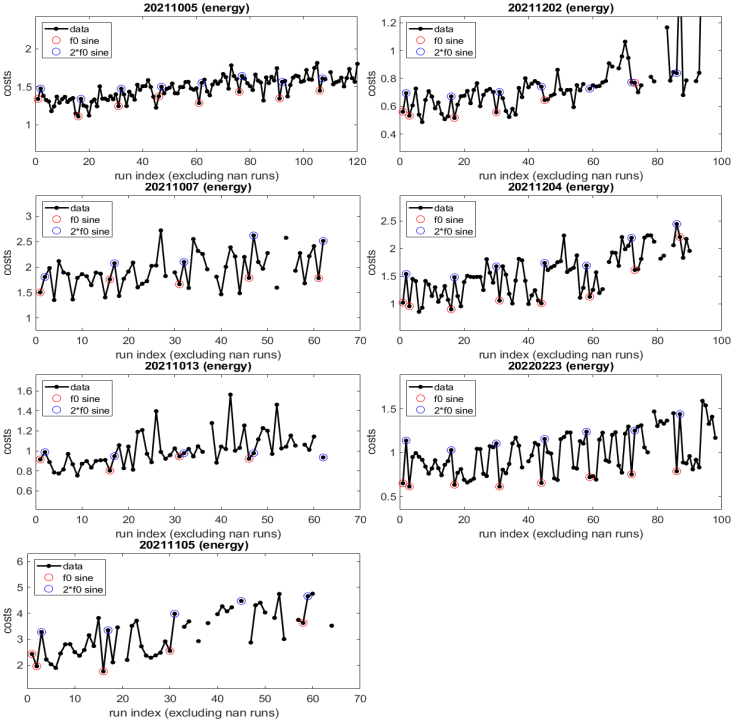


Additional file 1: Figure S10. Block thresholds in mA rms (“cost”) for all composite signal tests in bipolar cuffs. The circled data points were single sinusoids at the fundamental frequency (red) or at twice the fundamental frequency (blue).


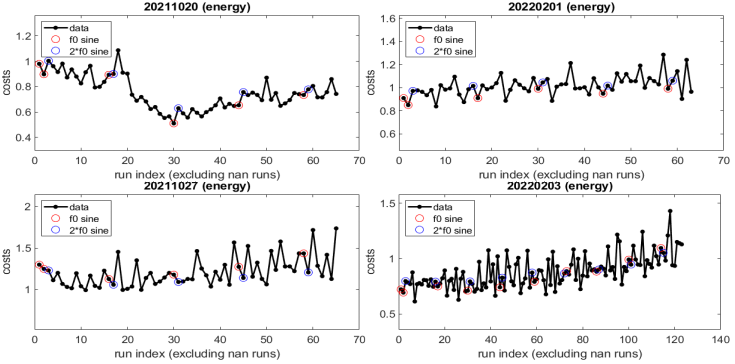


Additional file 1: Figure S11. Block thresholds in mA rms (“cost”) for all composite signal tests in tripolar cuffs. The circled data points were single sinusoids at the fundamental frequency (red) or at twice the fundamental frequency (blue).


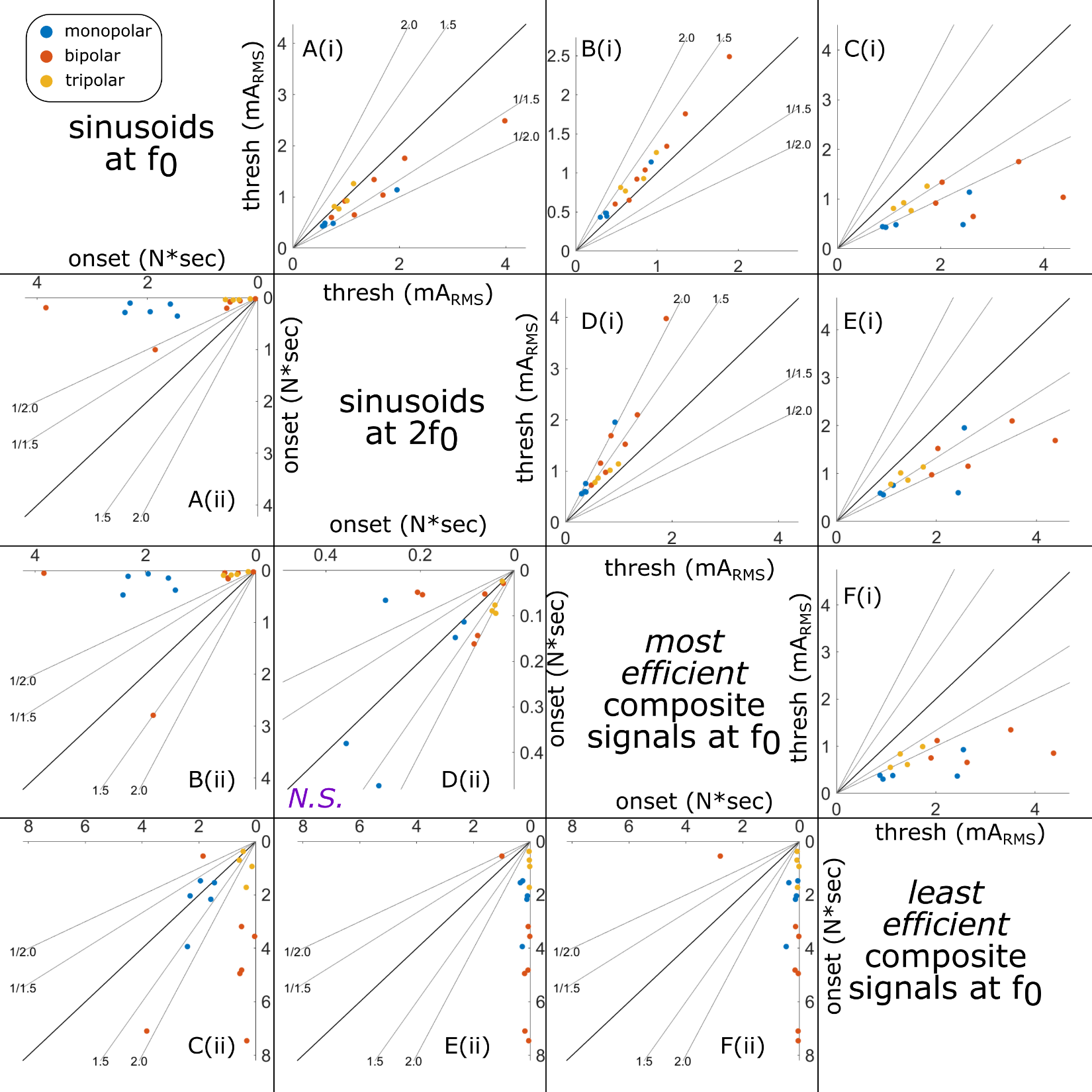


Additional file 1: Figure S12. Pairwise comparisons of thresholds and onset responses for f_0_, 2f_0_, and composite signals. Since f_0_ and 2f_0_ were measured multiple times throughout composite signal tests, the values shown are the median thresholds or the median onset responses of the replicates. No nerve underwent testing with multiple cuffs. All panels except the one with “N.S.” had significantly different pairwise comparisons by a signed rank test at α=0.05 after multiple comparison correction with Hochberg’s step-up procedure for twelve comparisons. Panels C(i), E(i), and F(i) exclude two outliers in which the least efficient composite signal did not block at tested amplitudes. For visualization, panel D(ii) excludes one data point in which the most efficient composite signal and the sinusoid at 2f_0_ produced an onset response of 2.8 and 1.0 N*sec (respectively). Consistent with previous studies, the *first harmonic* (i.e., sinusoid at f_0_) had overall lower block threshold (median: 0.8x; signed rank test p=1.7e-3) and a higher onset response (7x; p=4.4e-4) than the *second harmonic* (i.e., sinusoid at 2f_0_) (panels Ai, Aii), although thresholds for the first harmonic were not consistently lower than the second harmonic for the tripolar cuffs. The least efficient composite signals had higher threshold (2.4x; p=4.4e-4) and higher onset response (16x; p=1.1e-3) than the most efficient composite signal (panels Fi, Fii).


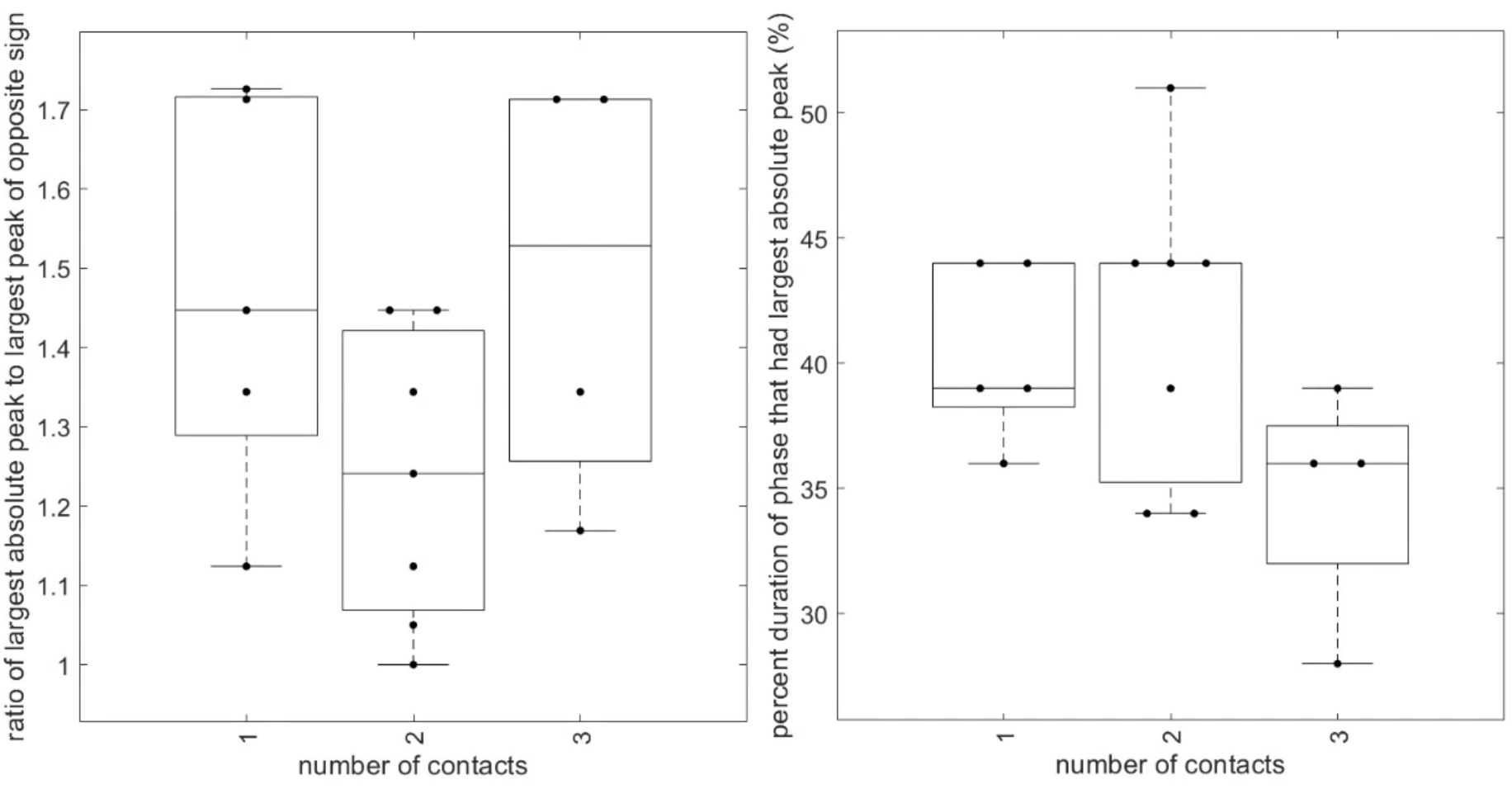


Additional file 1: Figure S13. Analysis of “monophasic nature” of most efficient waveforms in terms of ratio of peaks of opposing sign (left) and in terms of relative duration of the largest absolute peak (right). There was no clear difference between groups in these metrics (Kruskall-Wallis test: p = 0.19 (left); p = 0.20 (right)).


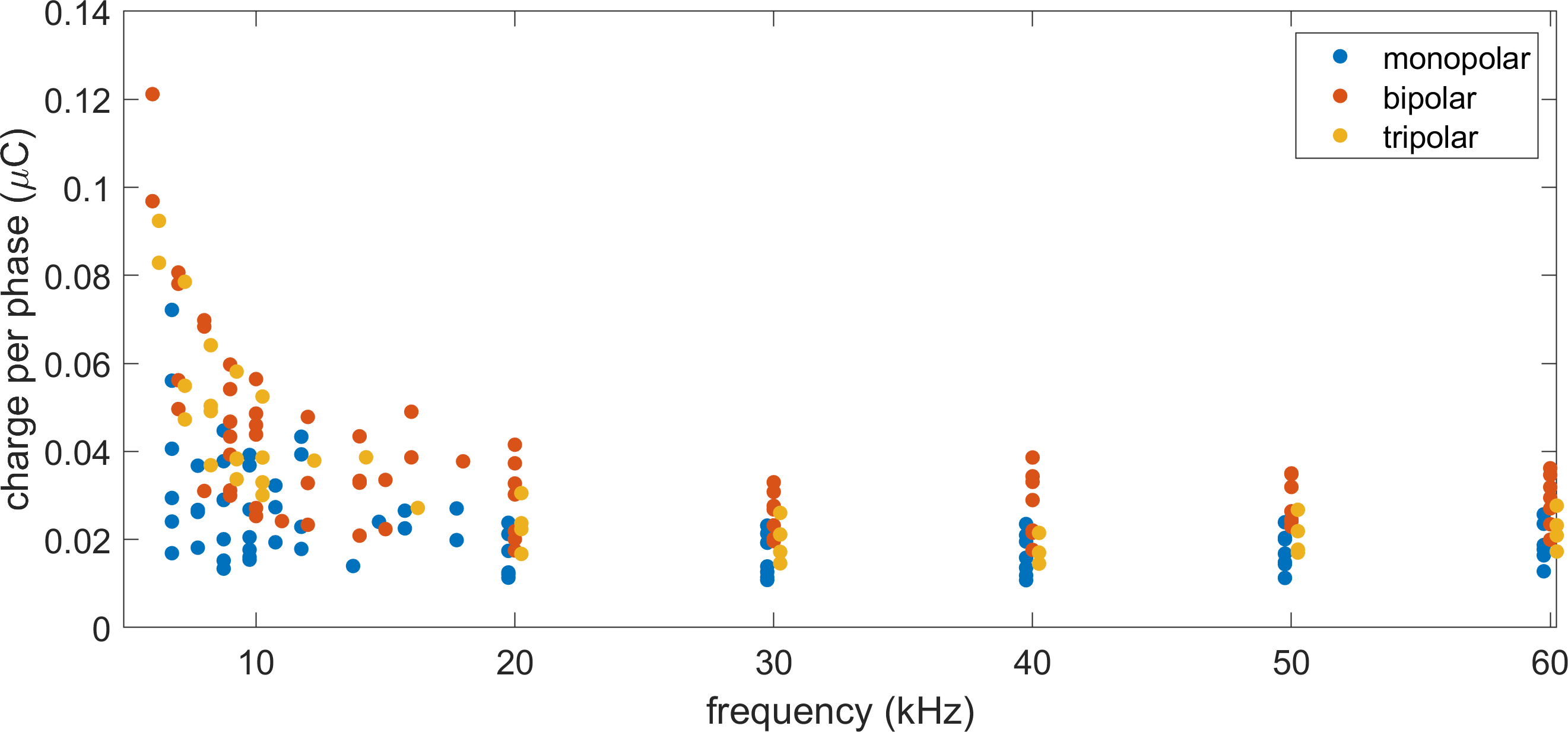


Additional file 1: Figure S14. Charge per phase for the same data shown in Figure 4A & B. The charge per phase was calculated as the block threshold current divided by (π*frequency).
